# Pulse dipolar EPR for determining nanomolar binding affinities†‡

**DOI:** 10.1039/d2cc02360a

**Published:** 2022-07-11

**Authors:** Katrin Ackermann, Joshua L. Wort, Bela E. Bode

**Affiliations:** EaStCHEM School of Chemistry, Biomedical Sciences Research Complex and Centre of Magnetic resonance, University of St Andrews, North Haugh St Andrews KY16 9ST Scotland UK beb2@st-andrews.ac.uk

## Abstract

Protein interaction studies often require very low concentrations and highly sensitive biophysical methods. Here, we demonstrate that pulse dipolar electron paramagnetic resonance spectroscopy allows measuring dissociation constants in the nanomolar range. This approach is appealing for concentration-limited biomolecular systems and medium-to-high-affinity binding studies, demonstrated here at 50 nanomolar protein concentration.

## Abstract 2

Cu^II^-nitroxide RIDME measurements at 100 nM protein concentration allow reliable extraction of dissociation constants and distances, while measurements at 50 nM protein concentration allow reliable extraction of dissociation constants only.

## Graphical abstract

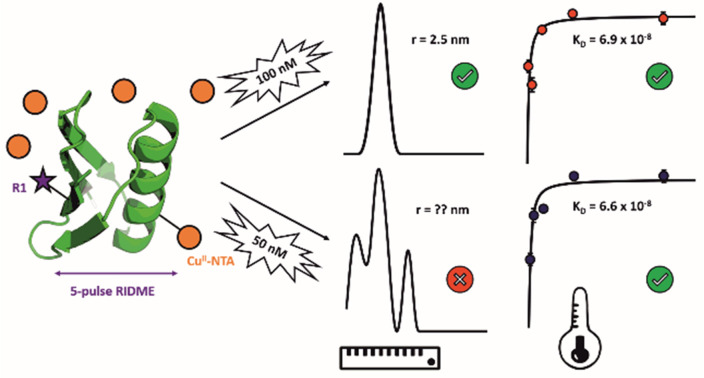

The study of biomacromolecular assemblies and protein–protein interactions in a physiologically relevant context often requires sub-micromolar concentrations of their constituents. Quantitative analyses in this concentration regime are a challenge for every magnetic resonance technique and require extraordinary sensitivity.

Pulse Dipolar electron paramagnetic resonance Spectroscopy (PDS) has gained reputation as a highly accurate biophysical method affording superb concentration sensitivity.^1–3^ PDS provides distance distributions derived from dipolar coupling frequencies between paramagnetic centres (Fig. 1A), usually introduced *via* site-directed spin-labelling with stable radicals such as nitroxides or trityls, or paramagnetic metal ions such as Gd^III^ or Cu^II^.^2,4–13^ Since PDS is exclusively sensitive to these paramagnetic centres, size or complexity of the biomolecular system are not a limiting factor, providing valuable long-range distance constraints from in-solution to in-cell environments.^3,5,14–26^

**Fig. 1:**
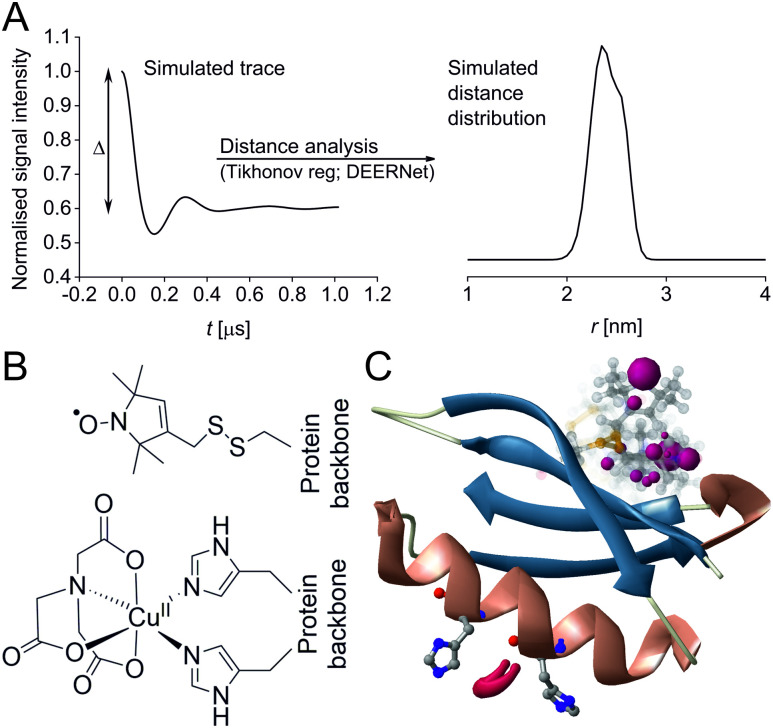
Predicted spatial distribution of the R1 label and Cu^II^-NTA in the GB1 mutant I6R1/K28H/Q32H based on crystal structure PDB 4WH4. (A) Simulated PDS trace with the modulation depth Δ indicated (left) and corresponding simulated distance distribution (right; MMM version 2021.2).^27,28^ (B) Structure drawing for the R1 label (top) and Cu^II^-NTA (bottom) coordinated to two histidine residues. (C) Visualisation of the modelled R1 rotamers (purple clouds show the position of the nitroxide group bearing the unpaired electron, with the cloud sized according to the probability of the population; rotamers are displayed using ball and stick representation) and the predicted spatial Cu^2+^ distribution between the two histidine residues (red shape visualises distribution of modelled Cu^2+^ positions).

Two of the most popular PDS methods are Pulsed ELectron–electron DOuble Resonance (PELDOR aka DEER for Double Electron–Electron Resonance)^29–31^ and Relaxation Induced Dipolar Modulation Enhancement (RIDME),^32,33^ with methodology and hardware being continuously developed.^34–39^ Furthermore, deep learning based neural network processing^40,41^ of PDS data is rapidly gaining interest as it overcomes potential confirmation bias by the user and it has been recommended as a standard processing approach.^42^

In recent years, the use of genetically encoded double-histidine (dHis) motifs to bind paramagnetic metal ions, in particular Cu^II^, has been established for PDS.^7,27,43–45^ Here, orthogonal labelling, that is, the combination of two spectroscopically distinct spin labels (in this case methanethiosulfonate (MTSL)^6,46^ and Cu^II^-nitrilotriacetic acid (Cu^II^-NTA),^43^Fig. 1B) with the RIDME method, has enabled PDS measurements in the sub-μM concentration regime.^47^ Nanomolar sensitivity for PDS could so far be demonstrated at 45 nM protein (90 nM spin) concentration using a new trityl spin label and a single-frequency method (double quantum coherence, DQC),^2,34^ and at ∼100 nM protein (200 nM spin) concentration using the spin label MTSL and PELDOR method.^1,3^ However, for the determination of dissociation constants (*K*_D_s) the orthogonal labelling approach is highly beneficial, as this allows preservation of the amplitude of the detected signal which encodes the dipolar coupling and binding site occupation (*via* the modulation depth Δ, illustrated in Fig. 1A) when ‘titrating’ one binding partner. Here, Cu^II^-NTA is added in increasing amounts to saturation. This technique for *K*_D_ determination is a direct approach similar to isothermal titration calorimetry (ITC),^48^ in contrast to secondary readouts such as native gel shift.^49^

Crucially, *K*_D_ determination requires concentrations of the interacting partners approximately in the same order of magnitude as the dissociation constant itself.^47,49^ Thus, the biophysical method used must be of sufficient sensitivity. While nanomolar PDS-based binding studies would be highly desirable to have as complementary technique to ITC, a proof-of-principle study is currently lacking.

Here, the practical concentration limit for PDS-based binding studies on orthogonally labelled protein is approached using the well-established spin labels MTSL^6,46^ and Cu^II^-NTA^43^ grafted on to the *Streptococcus sp.* Group G protein G, B1 domain (GB1), which has been extensively studied by EPR.^7,9,43–45,50^ We demonstrate that Cu^II^-nitroxide RIDME binding studies are feasible as low as 50 nM protein concentration, thereby pushing the limit by an order of magnitude compared to previous data.^47^ This makes this technique appealing for protein interaction studies in physiological context, especially for concentration-limited biomolecular systems and for determination of nanomolar binding affinities, typical for important biomolecular interactions such as host-pathogen interactions, formation of enzyme-substrate complexes, or affinities of monoclonal antibodies.^51–53^

Previously, Cu^II^-nitroxide RIDME measurements at 500 nM GB1 concentration yielded both, distance distributions and a dissociation constant.^47^ However, recent benchmarking of sensitivity limits^1^ suggests a further order of magnitude reduction in concentration may be feasible which is explored here.

A set of RIDME distance measurements was performed on the GB1 construct I6R1/K28H/Q32H (Fig. 1C) at 100 nM protein concentration with varying Cu^II^-NTA concentrations (100 nM to 8.1 μM). Data were processed and validated using Tikhonov regularisation in DeerAnalysis2015^54^ as described previously^1^ (for details see ESI‡). Modulation depths continuously increased with Cu^II^-NTA concentration, and all extracted distance distributions gave the predicted mean distance (Fig. 2A and B). The emerging alternative deep neural network processing (DEERNet^41^ with RIDME background model^40^ within DeerAnalysis2022) confirmed these results (see ESI‡ for details).

**Fig. 2:**
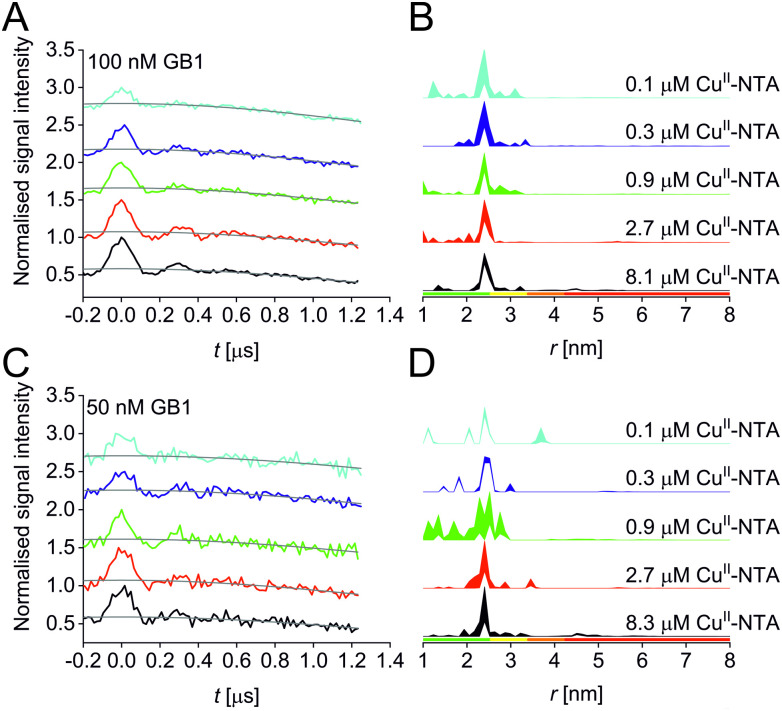
RIDME data for the 100 nM (top) and 50 nM (bottom) GB1 I6R1/K28H/Q32H pseudo-titration series. (A and C) Stacked raw RIDME traces (black) with background function (grey); (B and D) corresponding distance distributions given as 95% confidence estimates (±2*σ*) with 50% noise added for error estimation during statistical analysis. Colour bars represent reliability ranges (green: shape reliable; yellow: mean and width reliable; orange: mean reliable: red: no quantification possible).

Results suggest that 100 nM protein concentration is not yet the practical concentration limit. Therefore, protein concentration was further reduced to 50 nM GB1. Keeping the averaging times similar to the 100 nM experiments (i.e., about 60 hours per sample, which is approaching the economical and stability limits for measurements of this type), traces were expectedly about two-fold noisier, resulting in higher uncertainties in the distance distributions (Fig. 2C and D). Retrieved modulation depths were following the expected trend, and a repeat experimental set at 50 nM GB1 yielded comparable modulation depths, demonstrating robust data reproducibility even at very low protein concentrations (see ESI‡ for details). Notably, DEERNet analysis failed for six of the ten 50 nM samples, attributed to the comparably poor signal-to-noise ratios, which is in line with recommendations that data of this quality should no longer be used to analyse distance distributions.^42^

All three experimental sets were analysed with respect to binding affinities as described previously.^44,47^ Briefly, binding isotherms were generated considering experimental modulation depths as a function of Cu^II^-NTA concentrations to obtain respective dissociation constants (Fig. 3; see ESI‡ for details). Results are in good agreement between the different experimental sets, with *K*_D_ values in the nanomolar concentration range (10^−7^ to 10^−8^, a global *K*_D_ value was fitted to 7 × 10^−8^, see ESI‡), close to previous PDS results, and consistent with ITC experiments.^47^ Differences between the data points of the two 50 nM sets are within the 95% confidence range. This demonstrates the validity of the approach of using PDS for low-concentration protein interaction studies and the determination of nanomolar affinities.

**Fig. 3:**
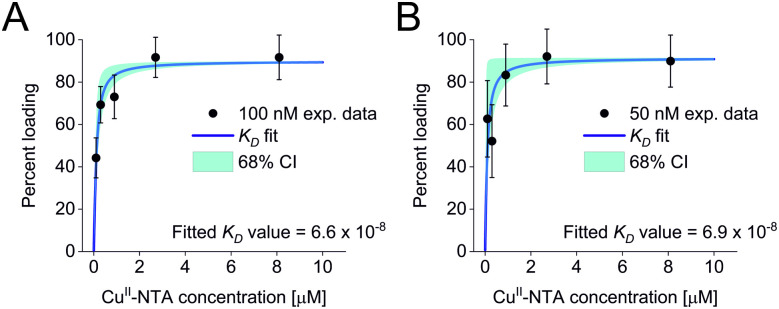
Binding isotherms for (A) 100 nM GB1 I6R1/K28H/Q32H and (B) 50 nM GB1 I6R1/K28H/Q32H. Fitted dissociation constants and 68% (1 × *σ*) confidence bounds (CI) are indicated on each plot.

Taken together, results demonstrate that both, binding studies and analysis of distance distributions are feasible down to 100 nM protein concentration at short to intermediate distances, whereas at 50 nM protein concentration modulation depths can still be obtained but under the present conditions, confident retrieval and interpretation of distance distributions is no longer possible due to the reduced signal-to-noise ratio,^42^ in the Cu^II^-nitroxide RIDME case.

PDS data were quantitatively assessed by comparing sensitivity values of the three experimental sets internally and with previous datasets (see ESI‡ for extensive analysis and discussion on sensitivities and tabulated values; see ref. 55 for the supporting research data†).^1,47^ Briefly, we define sensitivity as the ratio of the amplitude of the signal over experimental noise (or modulation-depth-to-noise ratio). As expected, the sensitivity of the 100 nM series is within error double the magnitude of the average sensitivity of the 50 nM series. Taking a recent quantification for the nitroxide–nitroxide PELDOR case^1^ into account, Cu^II^-nitroxide RIDME yields a comparable sensitivity but proves more robust towards extra losses at low concentration attributed to the more complex optimisation of PELDOR experiments (see ESI‡ for details).

In this study, we demonstrate that PDS is a suitable technique for low-concentration protein interaction studies and the determination of nanomolar binding affinities. Importantly, affinities can be directly derived from the modulation depth that encodes the fraction of complexes formed. Measurements are performed at much lower expense of materials than in standard ITC conditions^48^ (here, samples were measured at 100 or 50 nM protein concentration in a total volume of 65 μL). Furthermore, samples do not need to be immobilised as in surface plasmon resonance.^56^ In addition, spectroscopic orthogonality of spin labels provides a handle for tying affinities to certain spin pairs in more complex binding equilibria. This may be impossible to disentangle using other techniques that measure e.g., the aggregate heat of binding. Thus, PDS binding studies have significant potential to complement the ITC “gold standard”.

The ability to accurately determine binding affinities in the nanomolar range is relevant for e.g., the investigation of host-pathogen interactions such as collagen-binding bacterial surface proteins,^51^ or SARS-CoV2 antibody affinities for vaccine design.^53^ A recent survey of protein–protein complexes including antigen–antibody and enzyme-inhibitor interactions found the majority of complexes (90 out of 144) having *K*_D_s from 10^−6^ to 10^−10^,^52^ highlighting the significance of this range.

In conclusion, data presented here demonstrate that quantitative analysis of PDS modulation depths for extraction of dissociation constants is still feasible down to tens of nanomolar protein concentration in favourable cases. Importantly, this holds even when distance distributions are no longer reliably retrievable. This widens the scope of PDS applications to low-concentration, medium-to-high-affinity protein interaction studies yielding high sensitivity and accuracy. This has significant potential to impact investigations of biomolecular systems that are concentration-limited and therefore, currently out of reach using traditional biophysical methods.^49^

This research was funded, in whole or in part, by the Wellcome Trust (204821/Z/16/Z). A CC BY or equivalent license is applied to the Author Accepted Manuscript arising from this submission, in accordance with the grant's open access conditions. B. E. B. and K. A. acknowledge support by the Leverhulme Trust (RPG-2018-397). J. L. W. acknowledges support by the BBSRC DTP Eastbio. B. E. B. acknowledges equipment funding by BBSRC (BB/R013780/1 and BB/T017740/1). We thank the StAnD (St Andrews and Dundee) EPR group for long-standing support and in particular Dr El Mkami for assistance with PDS experiments.

## Conflicts of interest

There are no conflicts to declare.

## Supplementary Material

CC-058-D2CC02360A-s001
