# Correction: Pulse dipolar EPR for determining nanomolar binding affinities

**DOI:** 10.1039/d2cc90293a

**Published:** 2022-08-09

**Authors:** Katrin Ackermann, Joshua L. Wort, Bela E. Bode

**Affiliations:** EaStCHEM School of Chemistry, Biomedical Sciences Research Complex and Centre of Magnetic resonance, University of St Andrews, North Haugh, St Andrews KY16 9ST Scotland UK beb2@st-andrews.ac.uk

## Abstract

Correction for ‘Pulse dipolar EPR for determining nanomolar binding affinities’ by Katrin Ackermann *et al.*, *Chem. Commun.*, 2022, DOI: https://doi.org/10.1039/d2cc02360a.

The authors regret that the link provided in Ref. 55 was not displayed correctly in the original article (the link was correct in the footnote on the first page). The correct link is provided in the reference shown below as ref. 1.

The Royal Society of Chemistry apologises for these errors and any consequent inconvenience to authors and readers.

## Supplementary Material
