# Supplementary material for: Pulse dipolar EPR for determining nanomolar binding affinities
Source: Chem Commun (Camb). 2022 Jul 11;58(63):8790–3. doi: 10.1039/d2cc02360a (PMC9350988; doi:10.1039/d2cc02360a)
Supplement: CC-058-D2CC02360A-s001 [file CC-058-D2CC02360A-s001.pdf]

Electronic Supplementary Material (ESI) for Chemical Communications.
This journal is © The Royal Society of Chemistry 2022

Supporting Information

Pulse dipolar EPR for determining nanomolar binding affinities

Katrin Ackermann, Joshua L. Wort and Bela E. Bode*

EaStCHEM School of Chemistry, Biomedical Sciences Research Complex and Centre of Magnetic resonance, University of St Andrews, North Haugh, St Andrews, KY16 9ST, Scotland.

Corresponding author Email: beb2@st-andrews.ac.uk

Table of Contents

1) Experimental Methods

A. EPR Sample Preparation page S3

B. Pulse dipolar EPR Spectroscopy page S3

C. RIDME Data Processing and Analysis page S3

D. Simulations and Modelling page S4

2) Supplementary Results

A. Additional RIDME Data page S5

B. Modulation Depths and Sensitivities page S8

C. Binding Studies page S11

3) References page S14

4) Author Contributions page S15

5) CDA Reports page S15

1) Experimental Methods

A. EPR Sample Preparation

A construct of the immunoglobulin-binding B1 domain of group G streptococcal protein G (GB1) with one double-histidine motif and one cysteine residue (I6C/K28H/Q32H) was used in this study. Expression, purification, and spin labelling with MTSL and Cu^{II}-NTA for this construct has been described previously.[1]

For PDS experiments, samples with a final volume of 65 μL were prepared with varying protein and Cu^{II}-NTA concentrations in deuterated phosphate buffer (42.4 mM Na_2HPO_4, 7.6 mM KH_2PO_4, 150 mM NaCl, pH 7.4) and 50% (v/v) deuterated ethylene glycol (Deutero) for cryoprotection as described.[1] Samples were transferred to 3 mm quartz EPR tubes and immediately immersed in liquid nitrogen.

In total, three pseudo-titration series were prepared of five discrete samples each, one at 100 nM and two at 50 nM final protein concentration, respectively. Cu^{II}-NTA concentrations ranged from 0.1 to 8.1 μM. In addition, a control sample was prepared at 25 μM protein and 30 μM Cu^{II}-NTA concentration for dummy experiments (see below).

B. Pulse dipolar EPR Spectroscopy

Pulse Dipolar EPR Spectroscopy (PDS) was performed at 34 GHz (Q-band) frequency, operating on a Bruker ELEXSYS E580 spectrometer with second frequency option (E580-400U) and 3 mm cylindrical resonator (ER 5106QT2-2w in TE012 mode). A pulse travelling wave tube (TWT) amplifier (Applied Systems Engineering) with nominal output of 150 W was used for pulse amplification. Temperature was controlled using a cryogen-free variable temperature cryostat (Cryogenic Ltd) operating in the 3.5 K to 300 K temperature range.

5-pulse RIDME experiments[2] for the 50 and 100 nM pseudo-titration series were recorded with the pulse sequence ($\pi/2 - \tau_1 - \pi - (\tau_1 + t) - \pi/2 - T_{mix} - \pi/2 - (\tau_2 - t) - \pi - \tau_2$ – echo) at the field position corresponding to the maximum of the Cu^{II}-NTA field-swept spectrum with 8-step phase cycling, a τ_1 of 400 ns, a τ_2 of 1500 ns, a shot repetition time (SRT) of 30 ms, and a critically coupled resonator (high Q).

Measurements were recorded for ~60 hours on average at 30 K and with a short (reference) and a long mixing time (5 and 200 μs, respectively) to allow deconvolution of the traces as described previously.[1]

"Dummy" PELDOR and RIDME measurements (1 scan each) were recorded at 25 μM protein concentration (construct GB1 I6R1/K28H/Q32H) for sensitivity estimates as described previously.[1] In these experiments, all delays are set to be constant, shifting the entire sequence by the dipolar increment. The corresponding trace is thus only varied by thermal noise on the acquired echo. Dummy RIDME experiments were recorded at 30 K, SRT 30 ms, and both, with a critically coupled and an over-coupled resonator to determine the effect of high versus low Q on sensitivity. Dummy PELDOR experiments were performed based on the 4-pulse DEER[3-5] pulse sequence ($\pi/2(\nu_A) - \tau_1 - \pi(\nu_A) - (\tau_1 + t) - \pi(\nu_B) - (\tau_2 - t) - \pi(\nu_A) - \tau_2$ – echo) at 50 K as described previously,[6] with a frequency offset (pump – detection frequency) of +80 MHz (~3 mT) and SRT 5 ms; τ_1 of 400 ns, τ_2 of 1300 ns, and pulse lengths of 16 and 32 ns for $\pi/2$ and π detection, respectively.

C. PDS Data Processing and Analysis

RIDME experiments were analyzed using DeerAnalysis2015[7] as previously described.[8] Briefly, data were subjected to Tikhonov regularization using a homogeneous 6-dimensional background function followed by statistical analysis (validation tool) varying background start from 5 to 30% of the trace length in 8 trials and varying the background dimension from 3 to 6 in 7 trials. Resulting best-fit background start time and dimension were subsequently used as starting points for a second round of Tikhonov regularization followed by a second round of

statistical analysis, this time also including the addition of 50% random noise in 16 trials; one of the two 50 nM series required a modification: only 10% random noise was added in the second validation round (Fig. S3). Second round validation trials were pruned with a prune level of 1.15, where trials exceeding the root mean square deviation of the best fit by at least 15% are discarded. In all cases the regularization parameter α was chosen according to the L-curve criterion[9] and the goodness-of-fit.

Additionally, RIDME data were analysed with deep neural network processing employing DEERNet[10] (Spinach Rev 5662) with specified RIDME background[11,12] within DeerAnalysis2022 (downloaded 2 February 2022) and with the standalone ComparativeDeerAnalyzer (Spinach Rev 5501) without specific RIDME background for comparison.

D. Modelling

Predicted distance distribution was modelled based on the GB1 crystal structure PDB 4WH4.[13] An R1 moiety was introduced at residue 6 and Cu^{II}-NTA at residues 28 and 32. All modelling was done using MMM 2021.2,[14,15] assuming ambient temperature (298 K). The corresponding cartoon representation and predicted distance distribution are shown in Figure 1 of the main text.

2) Supplementary Results

A. Additional RIDME Data

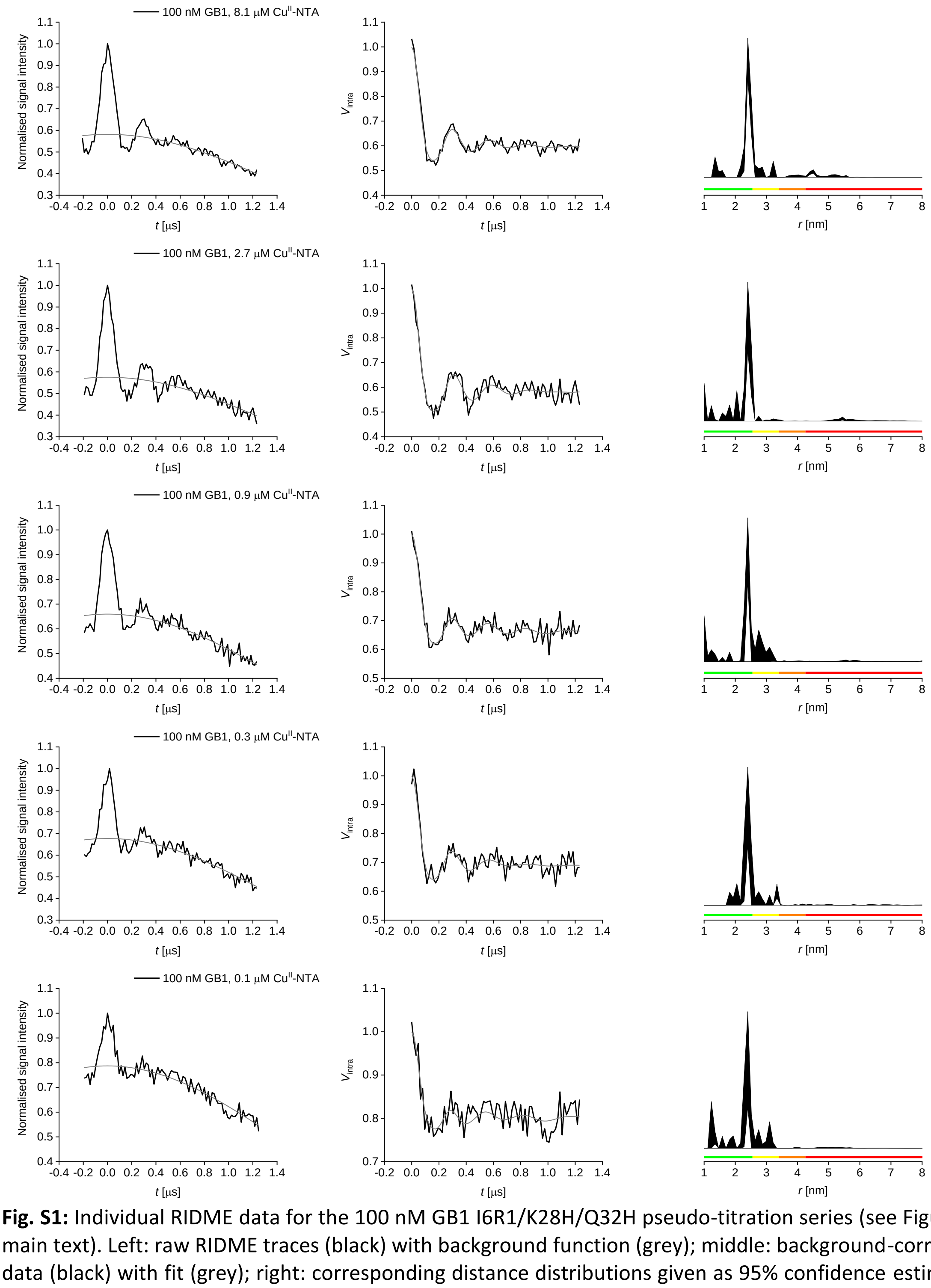

Fig. S1: Individual RIDME data for the 100 nM GB1 I6R1/K28H/Q32H pseudo-titration series (see Figure 2, main text). Left: raw RIDME traces (black) with background function (grey); middle: background-corrected data (black) with fit (grey); right: corresponding distance distributions given as 95% confidence estimates (± 2σ) with 50% noise added for error estimation during statistical analysis. Colour bars represent reliability ranges (green: shape reliable; yellow: mean and width reliable; orange: mean reliable: red: no quantification possible).

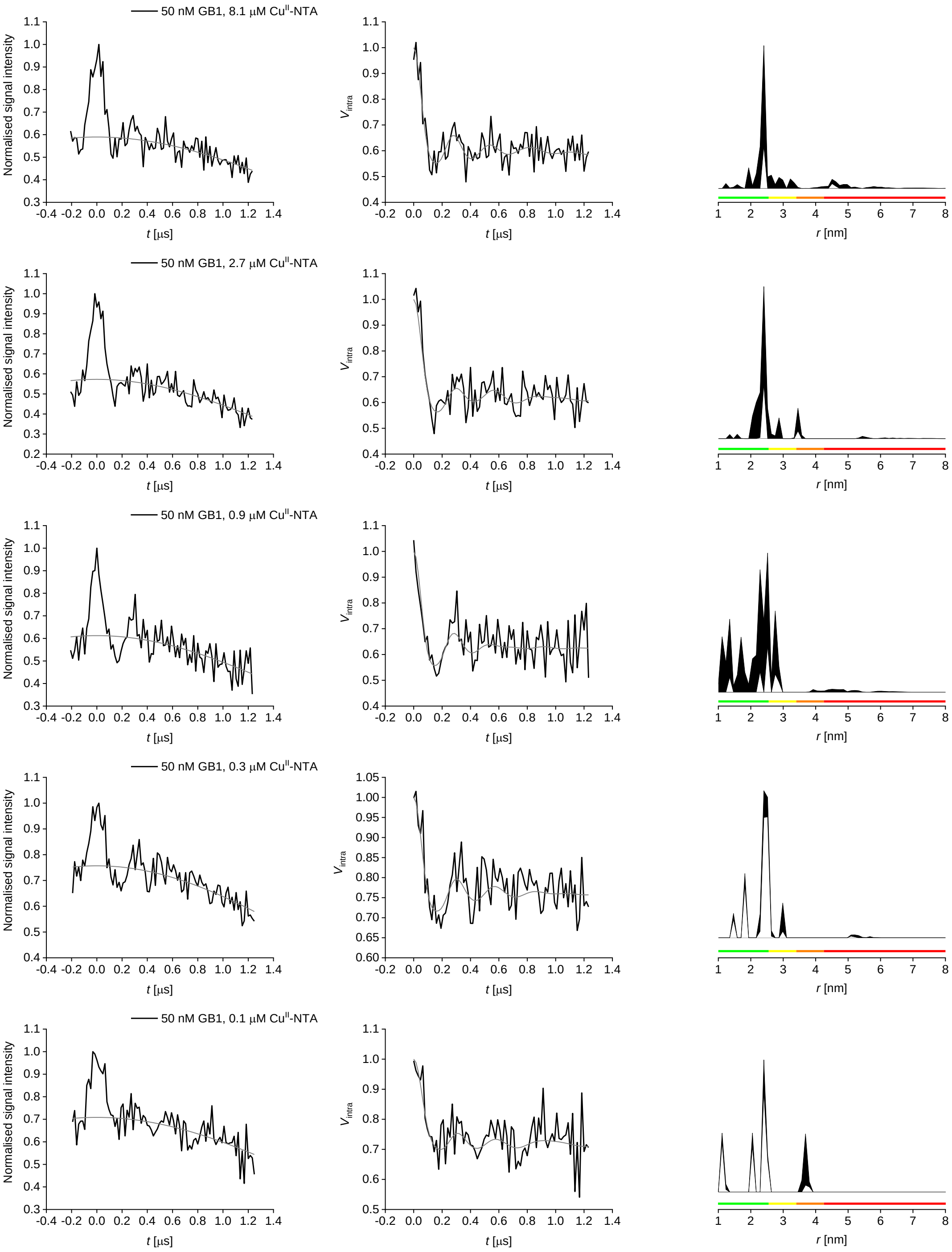

Fig. S2: Individual RIDME data for one of two 50 nM GB1 I6R1/K28H/Q32H pseudo-titration series (see Figure 2, main text). Left: raw RIDME traces (black) with background function (grey); middle: background-corrected data (black) with fit (grey); right: corresponding distance distributions given as 95% confidence estimates (± 2σ) with 50% noise added for error estimation during statistical analysis. Colour bars represent reliability ranges (green: shape reliable; yellow: mean and width reliable; orange: mean reliable: red: no quantification possible).

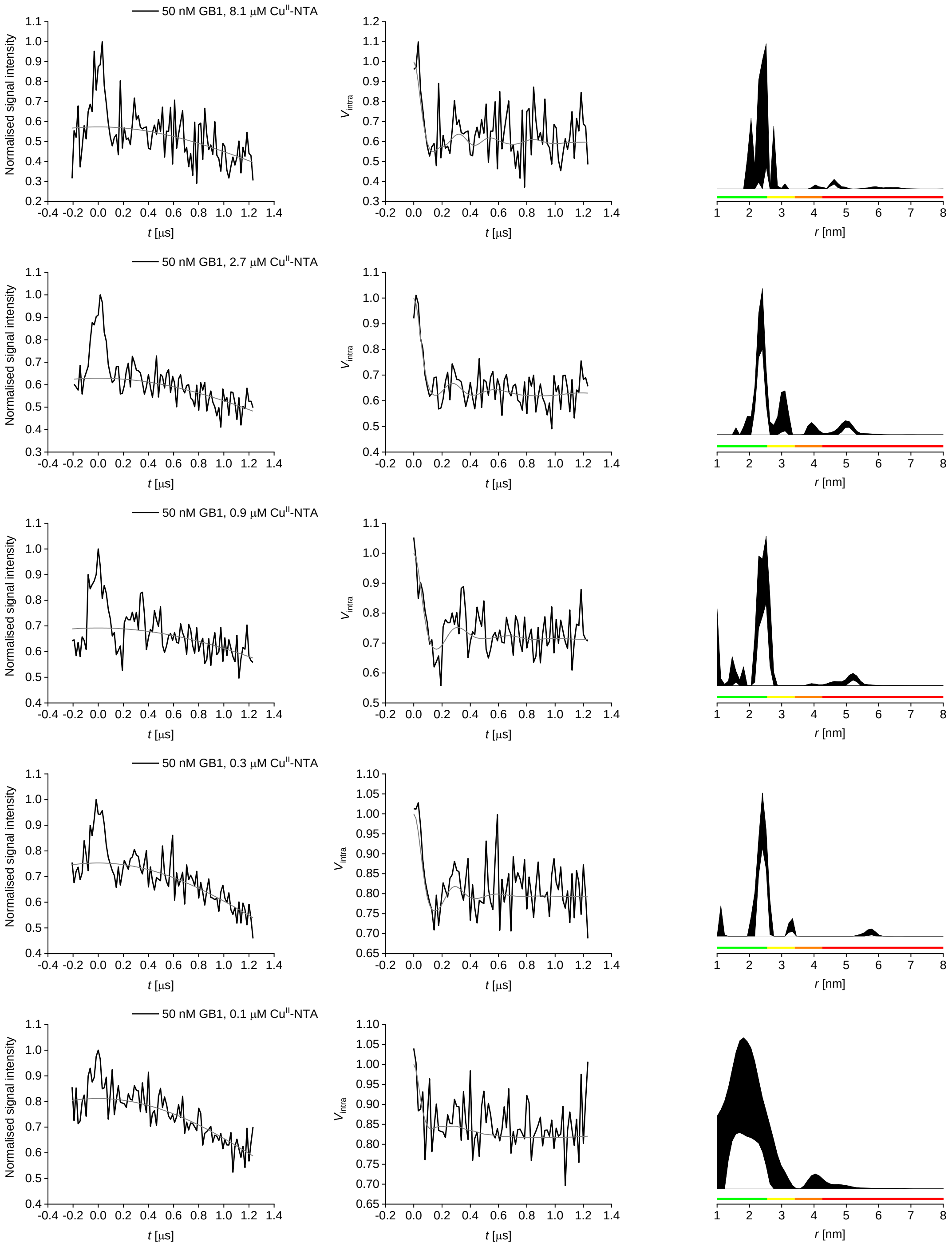

Fig. S3: Individual RIDME data for one of two 50 nM GB1 I6R1/K28H/Q32H pseudo-titration series. Left: raw RIDME traces (black) with background function (grey); middle: background-corrected data (black) with fit (grey); right: corresponding distance distributions given as 95% confidence estimates (± 2σ) with 10% noise added for error estimation during statistical analysis. Colour bars represent reliability ranges (green: shape reliable; yellow: mean and width reliable; orange: mean reliable: red: no quantification possible).

DEERNet analysis with RIDME background within DeerAnalysis2022 afforded results for all samples from the 100 nM pseudo-titration series, however for the lowest Cu^{II}-NTA concentration only the modulation depth was retrievable, not a distance distribution. For the two 50 nM pseudo-titration series, 6 out of 10 DEERNet analyses failed; we therefore refrain from using the remaining data sets here.

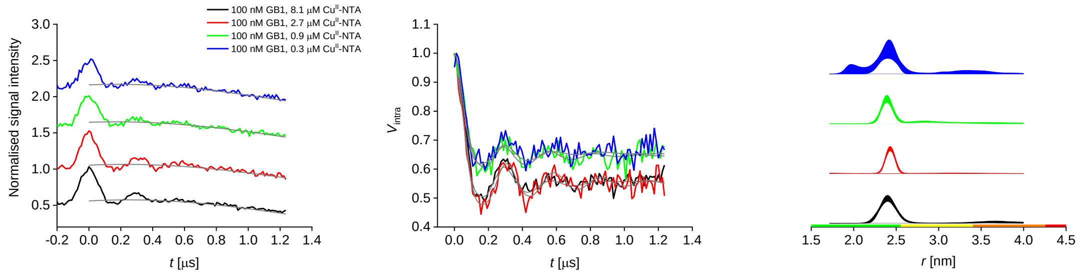

Fig. S4: DEERNet RIDME data obtained for 100 nM GB1 I6R1/K28H/Q32H pseudo-titration series. Left: stacked raw RIDME traces with background function (grey); middle: background-corrected data with fit (grey); right: corresponding distance distributions with error estimate. Colour bars represent reliability ranges (green: shape reliable; yellow: mean and width reliable; orange: mean reliable: red: no quantification possible).

RIDME data analysed with the standalone ComparativeDeerAnalyzer (Spinach Rev 5501) yielded reports for all but the lowest Cu^{II}-NTA concentration for the 100 nM pseudo-titration; for the two 50 nM series 6 out of 10 possible reports were obtained, and we therefore refrain from using the remaining data sets here. Full reports for the 100 nM series are available at the end of the ESI.

B. Modulation Depths and Sensitivities

RIDME modulation depths were obtained either during processing in DeerAnalysis from the second round of Tikhonov regularisation (i.e., using optimised background start time and background dimension, see section "PDS Data Processing and Analysis" for details), as well as from processing in DEERNet and CDA (100 nM only).

Calculated noise levels (root mean square deviation, RMSD, as estimated from the second and third quartile of the imaginary part of the phase-corrected RIDME trace) are used for sensitivity calculations as described previously[8] and detailed below.

Dummy PELDOR and RIDME traces were recorded for the GB1 I6R1/K28H/Q32H construct at 25 µM protein concentration (Table S1) to estimate experimental noise for a given setup. The total number of echoes per point for each trace was kept constant at 128 (four shots per point in a 2-step phase cycle for PELDOR and one shot per point in an 8-step phase cycle for RIDME).

Experiment	RMSD estimate	Relative noise
PELDOR, LQ	0.0055	3.41
RIDME, LQ	0.0029	1.79
RIDME, HQ	0.0016	1.00

Table S1: Estimated noise of dummy PELDOR and RIDME experiments.

Results suggest that critical coupling gains a factor of ~1.8 in the single frequency (RIDME) experiment, whereas off-resonance detection loses a factor of ~1.9 (PELDOR vs RIDME LQ), not considering actual sensitivities which are further depending on modulation depths and signal averaging (see below). Results reproduce the same qualitative trend as obtained previously for the GB1 I6R1/K28R1 construct.[8]

Sensitivities (S) were determined as the ratio of modulation depth (Δ), obtained from Tikhonov regularisation, to noise (RMSD). S values were further divided by the square root of total echoes per point and multiplied with the square root of the averaging rate, yielding the sensitivity per unit time (S_t).[8]

For the three independent titration series performed in this study, a summary of obtained modulation depths from different processing approaches (see section 1.C for details) and sensitivities S and S_t is given in Table S2 below. Differences in modulation depths obtained from the CDA versus DEERNet can be attributed to the different background models (see section 1C).

The average S_t for the 100 nM series is expected to be two times larger than the average S_t over the two 50 nM series. This is confirmed by experimental values for S_t (0.51 vs. 0.22, respectively) within error, suggesting some additional 'penalty' for reducing concentration to the point where optimising the experiment becomes negatively affected by the low signal, as observed previously.[8] However, this penalty is lower for RIDME (this study) than for PELDOR,[8] likely owing to the more straightforward process of setting up RIDME experiments.

The average sensitivity S (modulation-depth-to-noise ratio), a common parameter to assess PDS data quality, for the 100 nM pseudo-titration series was 16.0, while the average S for the two 50 nM series combined was 6.6. These findings are in line with recent recommendations that sensitivities substantially below 10 should not be used to analyse distance distributions.[16]

GB1 [nM]	Cu^{II} [μM]	RMSD	Δ Tikh.	Δ DEERNet	Δ CDA	S	S_t
50	8.1	0.084	0.427	0.428	0.471	5.11	0.17
50	2.7	0.045	0.371	0.344	0.456	8.25	0.27
50	0.9	0.052	0.308	n.a.	0.388	5.88	0.20
50	0.3	0.045	0.247	0.257	n.a.	5.48	0.18
50	0.1	0.041	0.188	n.a.	n.a.	4.57	0.15
50	8.1	0.053	0.410	n.a.	0.379	7.73	0.26
50	2.7	0.044	0.427	n.a.	n.a.	9.72	0.31
50	0.9	0.061	0.388	0.399	0.458	6.37	0.21
50	0.3	0.039	0.243	n.a.	0.257	6.19	0.20
50	0.1	0.047	0.292	n.a.	n.a.	6.28	0.21
100	8.1	0.018	0.418	0.440	0.380	23.78	0.79
100	2.7	0.024	0.425	0.446	0.409	17.75	0.59
100	0.9	0.022	0.340	0.354	0.298	15.71	0.43
100	0.3	0.023	0.323	0.337	0.303	14.27	0.45
100	0.1	0.025	0.206	0.235	n.a.	8.25	0.27

Table S2: Modulation depths Δ obtained for the three pseudo-titration series using the DeerAnalysis2015-based RIDME data processing with Tikhonov regularisation, DEERNet within DeerAnalysis2022, and the ComparativeDeerAnalyzer (CDA). Failed DEERNet and CDA runs where no modulation depth was obtained are indicated with "n.a.". Sensitivities S (modulation-depth-to-noise ratio) and sensitivities per unit time (S_t) were calculated from Δ Tikh.

For comparison of sensitivities between experiments and studies Table S3 summarises modulation depths and sensitivities per unit time S_t, extrapolated to a protein concentration of 1 μM. For extrapolation of sensitivities from dummy experiments it is necessary to assume modulation depths; here, values for Δ represent the nominal values obtained for fully labelled samples (0.2 for Cu^{II}-Cu^{II} RIDME; 0.3 for NO-NO PELDOR (here: hypothetical Cu^{II}-NO PELDOR); 0.01 for Cu^{II}-Cu^{II} PELDOR; 0.45 for Cu^{II}-NO RIDME). It should also be noted that for nitroxide-nitroxide PELDOR the factor 2 in S_t lost in low Q mode is compensated by the detection of two nitroxide labels instead of one, yielding an overall similar sensitivity (though with a larger 'optimisation penalty' at very low concentrations).[8]

GB1	Cu^{II}	Experiment	Δ	Δ	S_t	S_t at 1 μM	Ref.
[nM]	[μM]		Tikh.	assumed	experiment	extrapolated	
50	8.1	Cu^{II}-NO RIDME	0.427	n.a.	0.172	3.440	this study
50	2.7	Cu^{II}-NO RIDME	0.371	n.a.	0.269	5.376	this study
50	0.9	Cu^{II}-NO RIDME	0.308	n.a.	0.198	3.959	this study
50	0.3	Cu^{II}-NO RIDME	0.247	n.a.	0.182	3.650	this study
50	0.1	Cu^{II}-NO RIDME	0.188	n.a.	0.152	3.043	this study
50	8.1	Cu^{II}-NO RIDME	0.41	n.a.	0.257	5.147	this study
50	2.7	Cu^{II}-NO RIDME	0.427	n.a.	0.314	6.275	this study
50	0.9	Cu^{II}-NO RIDME	0.388	n.a.	0.205	4.110	this study
50	0.3	Cu^{II}-NO RIDME	0.243	n.a.	0.204	4.077	this study
50	0.1	Cu^{II}-NO RIDME	0.292	n.a.	0.207	4.135	this study
100	8.1	Cu^{II}-NO RIDME	0.418	n.a.	0.792	7.918	this study
100	2.7	Cu^{II}-NO RIDME	0.425	n.a.	0.591	5.909	this study
100	0.9	Cu^{II}-NO RIDME	0.340	n.a.	0.428	4.284	this study
100	0.3	Cu^{II}-NO RIDME	0.323	n.a.	0.452	4.516	this study
100	0.1	Cu^{II}-NO RIDME	0.206	n.a.	0.272	2.719	this study
25000	30	Dummy Cu^{II}-NO PELDOR	n.a.	0.300	68.414	2.737	this study
25000	30	Dummy Cu^{II}-NO RIDME LQ	n.a.	0.450	79.796	3.192	this study
25000	30	Dummy Cu^{II}-NO RIDME HQ	n.a.	0.450	143.009	5.720	this study
25000	50	Dummy Cu^{II}-Cu^{II} PELDOR	n.a.	0.010	0.658	0.026	[1]
25000	50	Dummy Cu^{II}-Cu^{II} RIDME HQ	n.a.	0.200	62.000	2.480	[1]
25000	30	Dummy Cu^{II}-NO RIDME HQ	n.a.	0.450	99.100	3.964	[1]
100	0	NO-NO PELDOR	0.223	n.a.	0.300	3.002	[8]
500	0	NO-NO PELDOR	0.292	n.a.	4.465	8.930	[8]
500	1.6	Cu^{II}-Cu^{II} RIDME	0.055	n.a.	0.452	0.905	[8]

Table S3: Modulation depths Δ and sensitivities per unit time (S_t) obtained for the three pseudo-titration series using the DeerAnalysis2015-based RIDME data processing with Tikhonov regularisation (see also Table S2), Δ and S_t values for dummy PELDOR and RIDME experiments, Cu^{II}-Cu^{II} RIDME and nitroxide-nitroxide PELDOR experiments. All S_t values are extrapolated to a protein concentration of 1 μM for direct comparison.

C. Binding Studies

Binding affinities for the pseudo-titration series based on experimental RIDME modulation depths (Δ) were determined as described previously.[1] Estimates for spin-lattice relaxation times (T_1) were determined previously for the Cu^{II}-NTA concentrations used here, assuming a mono-exponential relaxation behaviour (Table S4); in addition, data were fitted assuming a uniform T_1 of 50 μs for comparison of the relative stability of the determined dissociation constants (K_D). The mixing time T_{mix} and T_1 dependent modulation depth (Δ_{Tmix}) was calculated as described.[1] The ratio Δ/Δ_{Tmix} is given as 'percentage loading' in the main text (Figure 3). The 68% confidence interval (CI) bounds are taken as the binding isotherms simulated with the fitted K_D value ± σ, except for the 50 nM (I) series assuming uniform T_1, where this would result in a negative and thus, unphysical K_D. Therefore, in this case a tighter binding was assumed, with the affinity upper bound simulated as 6.3×10^{-11} and the affinity lower bound simulated as 1.38×10^{-7}. Here, σ is calculated as the standard deviation of the fitted Gaussian to the one-dimensional error surfaces (shown in figure S8).

Cu^{II}-NTA conc. [μM]	Mono-exponential T_1 [μs]	T_1 error [μs]
0.1	47.8	1.92
0.3	44.3	1.26
0.9	41.5	0.69
2.7	36.6	0.46
8.1	28.6	0.21

Table S4: Previously determined estimates for T_1 relaxation times, taken from Wort *et al.*[1]

Results of the binding studies are highly consistent regardless of the T_1 used for fitting:

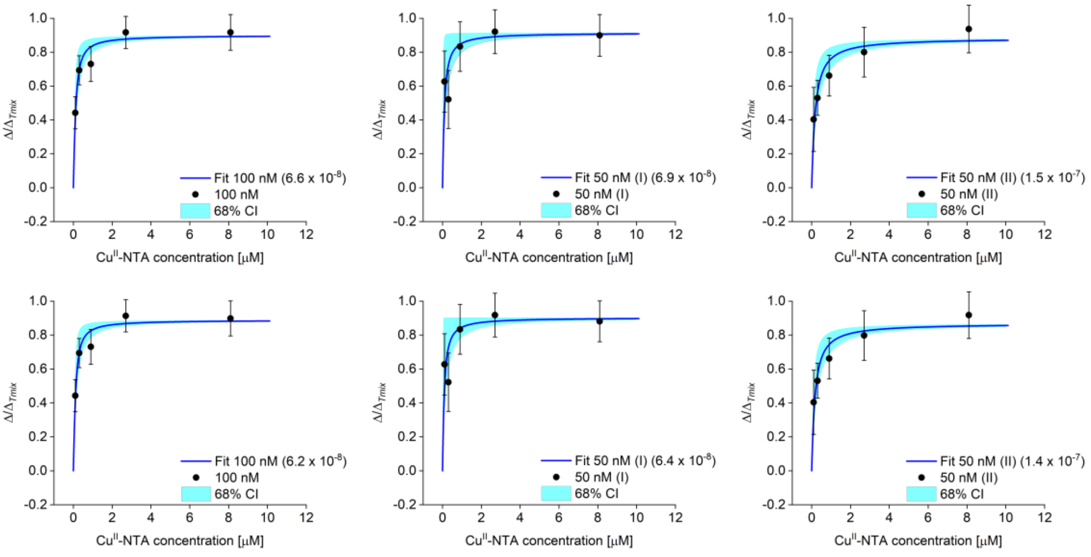

Fig. S5: Binding isotherms. Bivariate fits of K_D and Δ_{Tmix} are given for the individual pseudo-titration series assuming either previously determined T_1 estimates (top row) or uniform T_1 (bottom row). Error bars in the isotherm plots are ± 2 × error in $\Delta \times (\Delta_{Tmix})^{-1}$; T_1 errors are given in Table S4, for error analysis with uniform T_1 the highest empirically obtained error (1.92 μs) was applied for all data points. Fitted K_D values and 68% (1 × σ) confidence bounds (CI) for individual series are given in parentheses on each plot.

Binding isotherms for the two 50 nM series exhibit some differences due to larger errors during sample preparation (e.g., resulting from higher uncertainties in protein concentration

determination), however fits including T_1 errors show that the differences are still within the 95% confidence range.

Error contour plots for the 100 nM and one of the 50 nM pseudo-titration series illustrate the high reproducibility of the results albeit the larger error at lower concentration, demonstrating the validity of the approach to use PDS for low-concentration protein interaction studies:

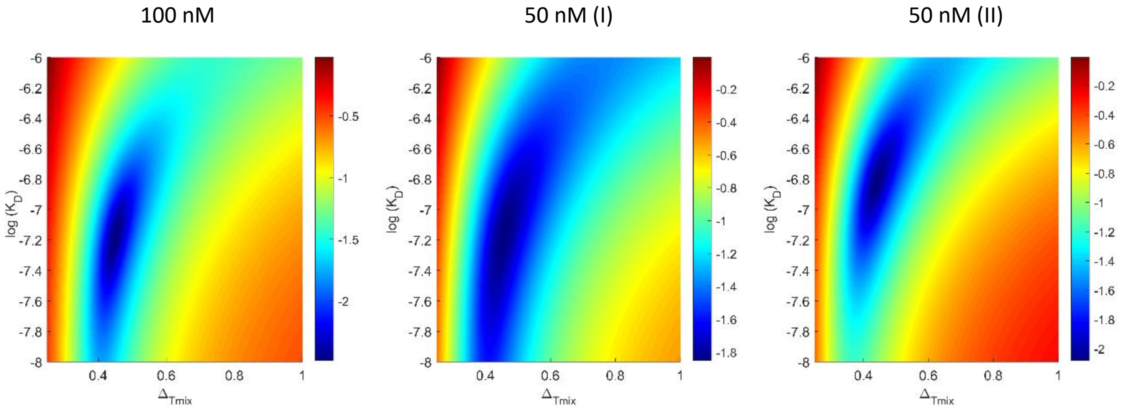

Fig. S6: Error contour plots corresponding to binding isotherms assuming previously determined T_1 estimates (top row, right Fig. S5). Left: 100 nM, middle: 50 nM (I), right: 50 nM (II) series. The error contours have a log K_D axis and log intensity (Z-dimension) axis.

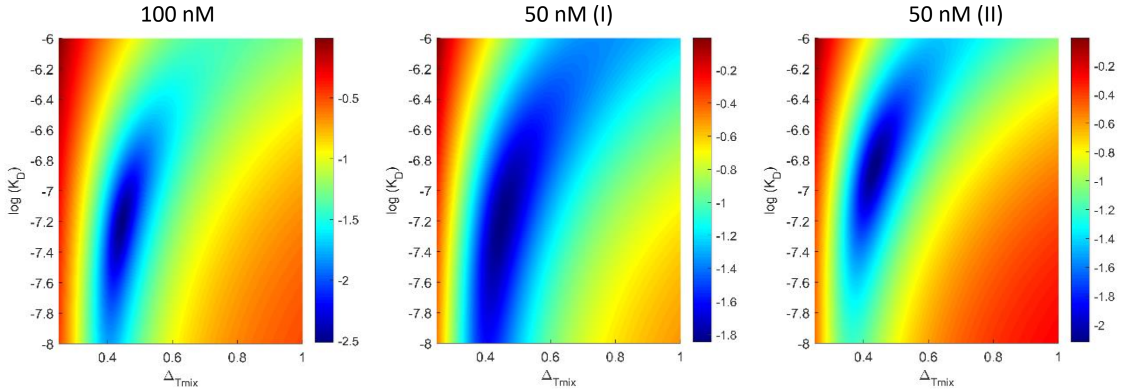

Fig. S7: Error contour plots corresponding to binding isotherms assuming uniform T_1 (see bottom row Fig. S5). Left: 100 nM, middle: 50 nM (I), right: 50 nM (II) series. The error contours have a log K_D axis and log intensity (Z-dimension) axis.

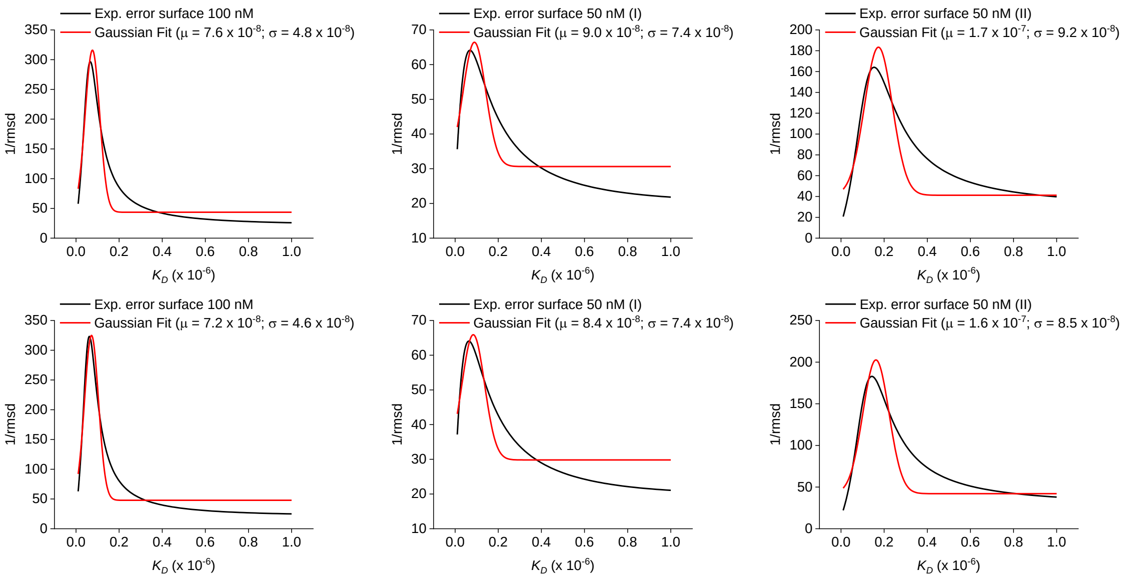

Fig. S8: Reciprocal experimental 1-dimensional error surfaces (black) and Gaussian fits (red) with mean (μ) and error (σ) of the K_D estimate. Experimental and fitted data are given for the individual pseudo-titration series assuming either previously determined T_1 estimates (top row) or uniform T_1 (bottom row).

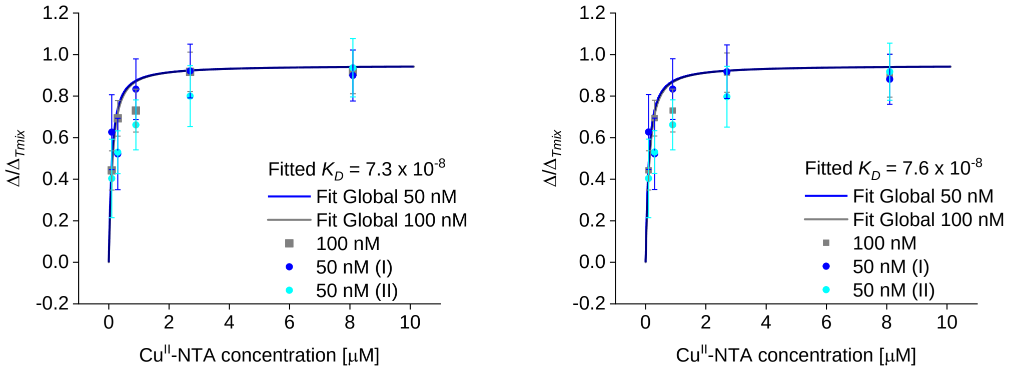

Fig. S9: Global K_D fits for the three pseudo-titration series assuming either previously determined T_1 estimates (left) or uniform T_1 (right).

3) References

1. J. L. Wort, K. Ackermann, A. Giannoulis, A. J. Stewart, D. G. Norman and B. E. Bode, *Angew. Chem. Int. Ed.*, 2019, **58**, 11681-11685.
2. S. Milikisyants, F. Scarpelli, M. G. Finiguerra, M. Ubbink and M. Huber, *J. Magn. Reson.*, 2009, **201**, 48-56.
3. R. G. Larsen and D. J. Singel, *J. Chem. Phys.*, 1993, **98**, 5134-5146.
4. A. D. Milov, K. M. Salikov and M. D. Shirov, *Fiz. Tverd. Tela*, 1981, **23**, 975-982.
5. M. Pannier, S. Veit, A. Godt, G. Jeschke and H. W. Spiess, *J. Magn. Reson.*, 2000, **142**, 331-340.
6. P. S. Kerry, H. L. Turkington, K. Ackermann, S. A. Jameison and B. E. Bode, *J. Phys. Chem. B*, 2014, **118**, 10882-10888.
7. G. Jeschke, V. Chechik, P. Ionita, A. Godt, H. Zimmermann, J. Banham, C. R. Timmel, D. Hilger and H. Jung, *Appl. Magn. Reson.*, 2006, **30**, 473-498.
8. K. Ackermann, J. L. Wort and B. E. Bode, *J. Phys. Chem. B*, 2021, **125**, 5358-5364.
9. Y. W. Chiang, P. P. Borbat and J. H. Freed, *J. Magn. Reson.*, 2005, **172**, 279-295.
10. S. G. Worswick, J. A. Spencer, G. Jeschke and I. Kuprov, *Sci. Adv.*, 2018, **4**, eaat5218.
11. J. Keeley, T. Choudhury, L. Galazzo, E. Bordignon, A. Feintuch, D. Goldfarb, H. Russell, M. J. Taylor, J. E. Lovett, A. Eggeling, L. Fábregas Ibáñez, K. Keller, M. Yulikov, G. Jeschke and I. Kuprov, *J. Magn. Reson.*, 2022, **338**, 107186.
12. K. Keller, M. Qi, C. Gmeiner, I. Ritsch, A. Godt, G. Jeschke, A. Savitsky and M. Yulikov, *Phys. Chem. Chem. Phys.*, 2019, **21**, 8228-8245.
13. T. F. Cunningham, M. R. Putterman, A. Desai, W. S. Horne and S. Saxena, *Angew. Chem. Int. Ed.*, 2015, **54**, 6330-6334.
14. S. Ghosh, S. Saxena and G. Jeschke, *Appl. Magn. Reson.*, 2018, **49**, 1281-1298.
15. Y. Polyhach, E. Bordignon and G. Jeschke, *Phys. Chem. Chem. Phys.*, 2011, **13**, 2356-2366.
16. O. Schiemann, C. A. Heubach, D. Abdullin, K. Ackermann, M. Azarkh, E. G. Bagryanskaya, M. Drescher, B. Endeward, J. H. Freed, L. Galazzo, D. Goldfarb, T. Hett, L. Esteban Hofer, L. Fábregas Ibáñez, E. J. Hustedt, S. Kucher, I. Kuprov, J. E. Lovett, A. Meyer, S. Ruthstein, S. Saxena, S. Stoll, C. R. Timmel, M. Di Valentin, H. S. McHaourab, T. F. Prisner, B. E. Bode, E. Bordignon, M. Bennati and G. Jeschke, *J. Am. Chem. Soc.*, 2021, **143**, 17875-17890.

4) Author Contributions

Katrin Ackermann: Conceptualisation (equal), data curation (lead), formal analysis (lead), funding acquisition (supporting), investigation (lead), writing-original draft (lead), writing-review & editing (equal)

Joshua L. Wort: Conceptualisation (supporting), formal analysis (supporting), investigation (supporting), methodology (lead), software (lead), writing-original draft (supporting), writing-review & editing (equal)

Bela E. Bode: Conceptualisation (equal), investigation (supporting), formal analysis (supporting), funding acquisition (lead), methodology (supporting), supervision (lead), writing-original draft (supporting), writing-review & editing (equal)

5) CDA Reports

In the following the full CDA reports obtained for the 100 nM series are attached.

Cu^{II} [μM] for 100 nM GB1 I6R1/K28H/Q32H	Code (dataset name for CDA report)
8.1	190419_KAq104.3_RIDME_5_200_200000_by_5000
2.7	190531_KAq105.3_RIDME_5_200_200000_by_5000
0.9	190615_KAq108.3_RIDME_5_200_200000_by_5000
0.3	190609_KAq107.3_RIDME_5_200_200000_by_5000

DEER analysis report on dataset 190419_KAq104.3_RIDME_5_200_200000_by_5000

DEERNet Spinach SVN Rev 5501 and DeerLab 0.9.1 Tikhonov regularization

ComparativeDEERAnalyzer

see: S. G. Worswick et al., DOI: 10.1126/sciadv.aat5218, L. Fabregas Ibanez et al., DOI: 10.5194/mr-1-209-2020

01-Feb-2022 14:48:15

1. Distance distributions

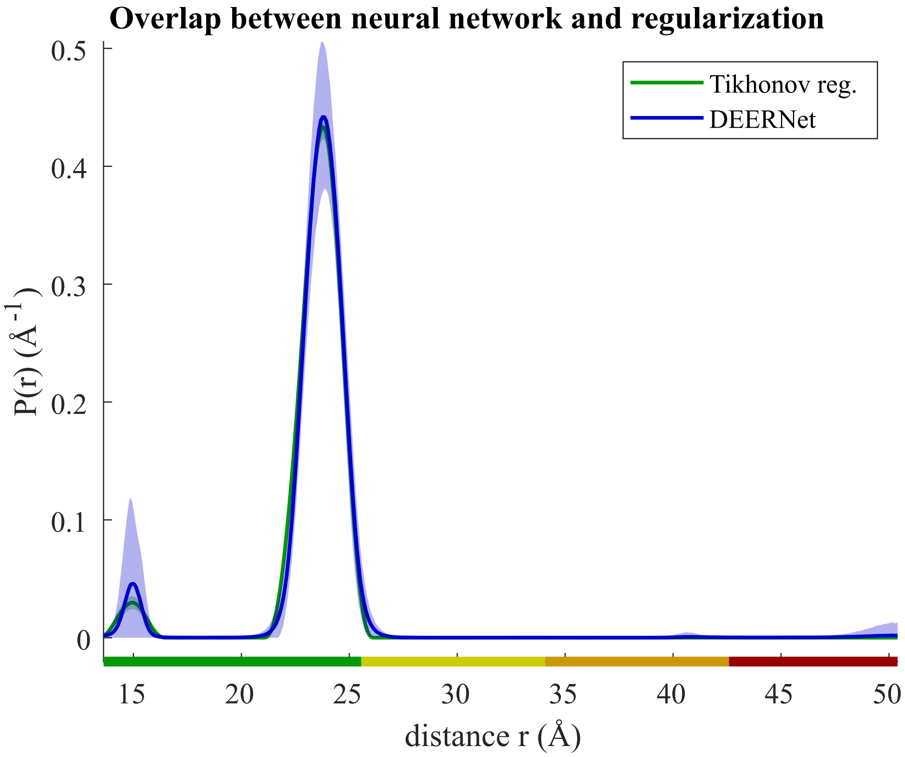

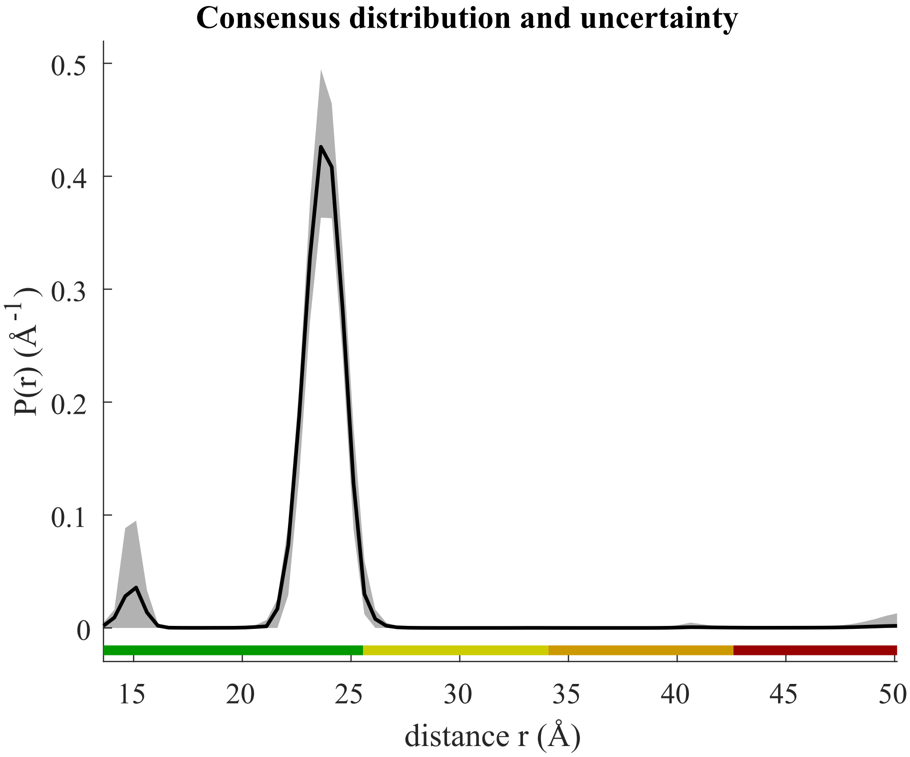

2. Fits of time-domain data

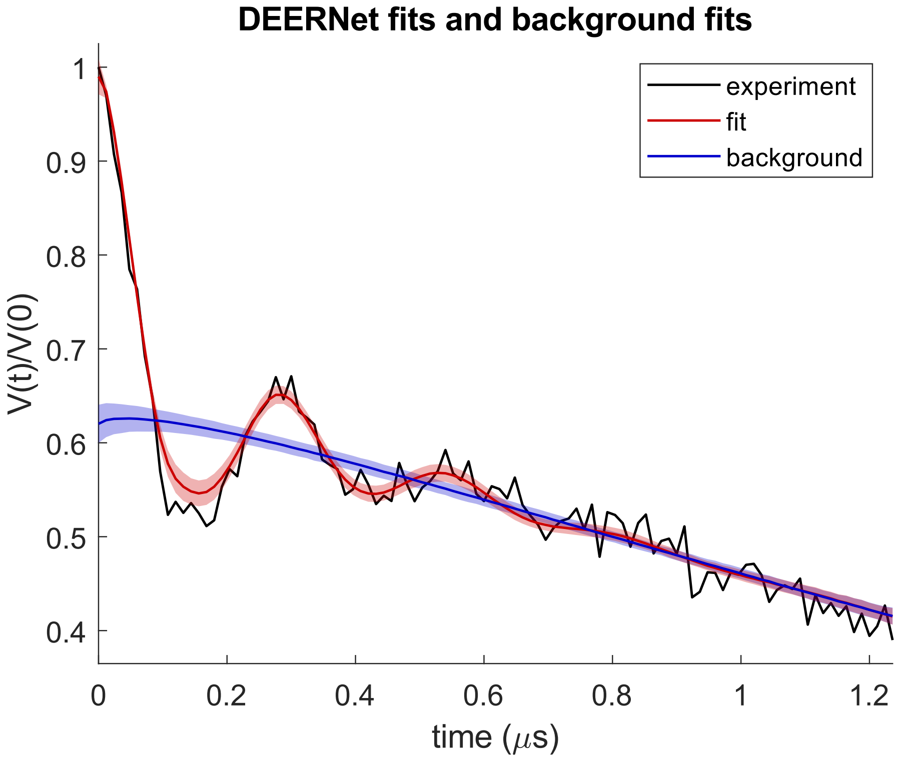

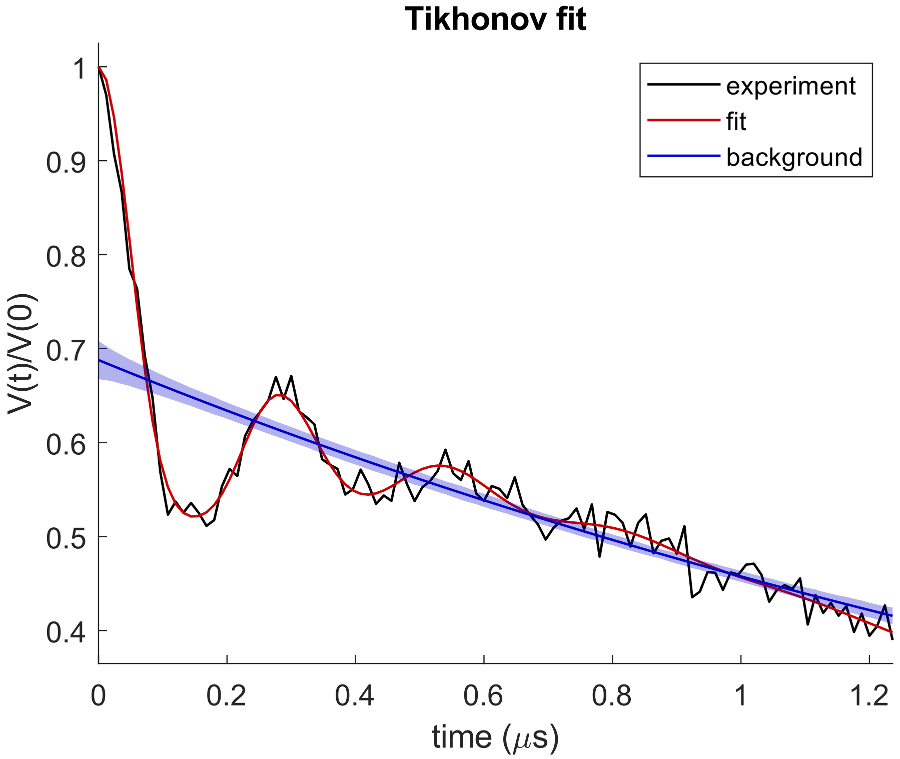

3. Experimental and processing parameters

DEERNet background not maximum at zero time. Long distances very uncertain.

Time increment: 12 ns

Maximum time: 1236 ns

Zero time: 8 ns

Phase: 0.0 degree

Noise estimates normalized to maximum signal

From imaginary part: 0.01681

From DEERNet fit: 0.01800

From Tikhonov fit: 0.01572

Modulation depth: 0.380

Signal-to-noise ratio: 21.1 (w.r.t. modulation)

Ensemble of 24 neural networks

Background separation by neural network

Regularization parameter by best overlap with neural network solution

Regularization parameter used: 1.25

Reg. par. initial estimate by L-curve corner: 5.01

Overlap between DEERNet and regularization solutions: 0.952

Mean distance: 23.7 Å

Distance standard deviation: 0.5 Å

Full data set in Matlab format: C:\Users\ka44\Documents\OneDrive - University of St Andrews\StAndrews\Work\BEB\Projects\GB1\GB1_Nanomolar\GB1_nM_data\Cu_NO\Titrations_Processing_2022\CDA\100nM_190411\190419_KAq104.3_RIDME_5_200_200000_by_5000_consensus_DEER_analysis.mat

Distance distributions in text format: C:\Users\ka44\Documents\OneDrive - University of St Andrews\StAndrews\Work\BEB\Projects\GB1\GB1_Nanomolar\GB1_nM_data\Cu_NO\Titrations_Processing_2022\CDA\100nM_190411\190419_KAq104.3_RIDME_5_200_200000_by_5000_consensus_DEER_distribution.dat

Fit and background in text format: C:\Users\ka44\Documents\OneDrive - University of St Andrews\StAndrews\Work\BEB\Projects\GB1\GB1_Nanomolar\GB1_nM_data\Cu_NO\Titrat

ions_Processing_2022\CDA\100nM_190411\190419_KAq104.3_RIDME_5_200_200000_by_5000_consensus_DEER_fit.dat

DEER analysis report on dataset 190531_KAq105.3_RIDME_5_200_200000_by_5000

DEERNet Spinach SVN Rev 5501 and DeerLab 0.9.1 Tikhonov regularization

ComparativeDEERAnalyzer

see: S. G. Worswick et al., DOI: 10.1126/sciadv.aat5218, L. Fabregas Ibanez et al., DOI: 10.5194/mr-1-209-2020

01-Feb-2022 15:08:41

1. Distance distributions

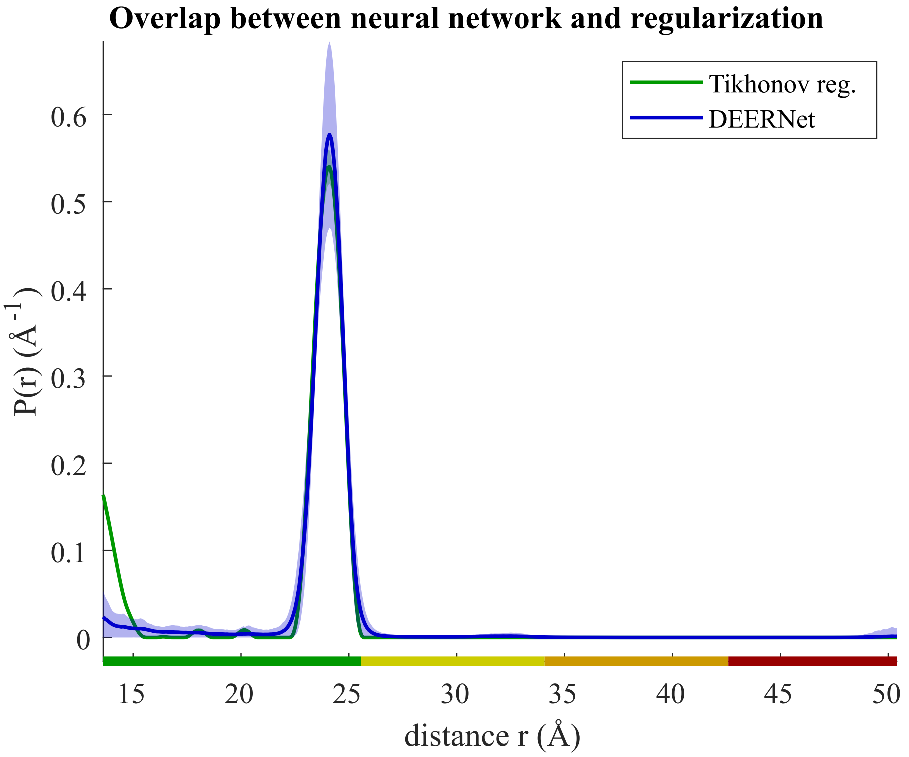

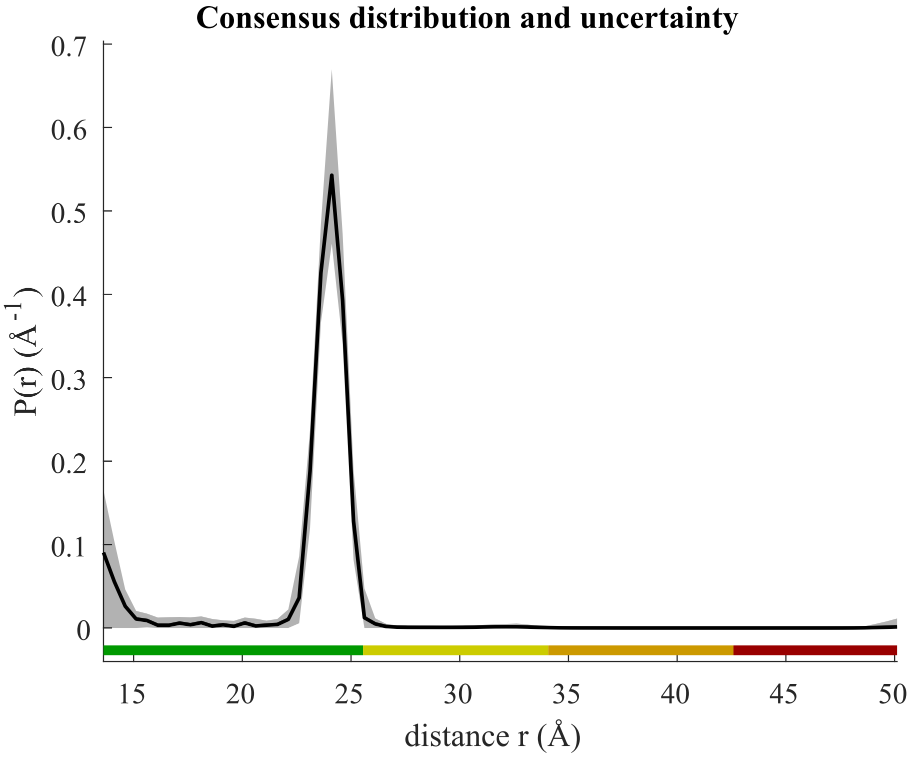

2. Fits of time-domain data

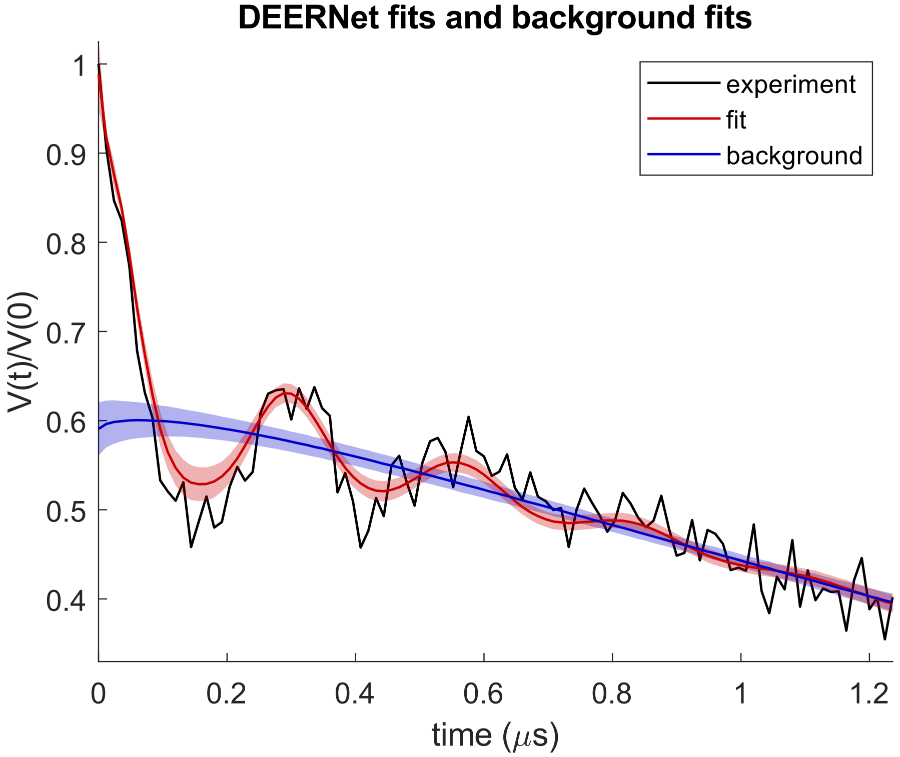

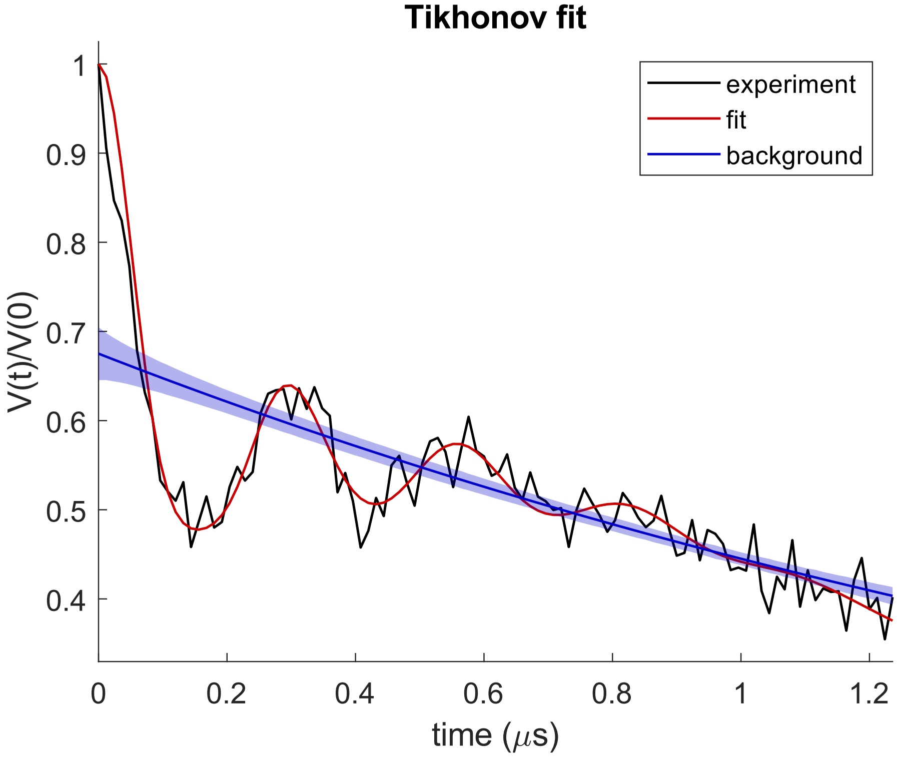

3. Experimental and processing parameters

DEERNet background not maximum at zero time. Long distances very uncertain.

Time increment: 12 ns

Maximum time: 1236 ns

Zero time: 7 ns

Phase: -0.3 degree

Noise estimates normalized to maximum signal

From imaginary part: 0.02336

From DEERNet fit: 0.02868

From Tikhonov fit: 0.02726

Modulation depth: 0.409

Signal-to-noise ratio: 14.3 (w.r.t. modulation)

Ensemble of 24 neural networks

Background separation by neural network

Regularization parameter by best overlap with neural network solution

Regularization parameter used: 0.50

Reg. par. initial estimate by L-curve corner: 3.98

Overlap between DEERNet and regularization solutions: 0.887

Mean distance: 23.9 Å

Distance standard deviation: 0.7 Å

Full data set in Matlab format: C:\Users\ka44\Documents\OneDrive - University of St Andrews\StAndrews\Work\BEB\Projects\GB1\GB1_Nanomolar\GB1_nM_data\Cu_NO\Titrations_Processing_2022\CDA\100nM_190411\190531_KAq105.3_RIDME_5_200_200000_by_5000_consensus_DEER_analysis.mat

Distance distributions in text format: C:\Users\ka44\Documents\OneDrive - University of St Andrews\StAndrews\Work\BEB\Projects\GB1\GB1_Nanomolar\GB1_nM_data\Cu_NO\Titrations_Processing_2022\CDA\100nM_190411\190531_KAq105.3_RIDME_5_200_200000_by_5000_consensus_DEER_distribution.dat

Fit and background in text format: C:\Users\ka44\Documents\OneDrive - University of St Andrews\StAndrews\Work\BEB\Projects\GB1\GB1_Nanomolar\GB1_nM_data\Cu_NO\Titrat

ions_Processing_2022\CDA\100nM_190411\190531_KAq105.3_RIDME_5_200_200000_by_5000_consensus_DEER_fit.dat

DEER analysis report on dataset 190615_KAq108.3_RIDME_5_200_200000_by_5000

DEERNet Spinach SVN Rev 5501 and DeerLab 0.9.1 Tikhonov regularization

ComparativeDEERAnalyzer

see: S. G. Worswick et al., DOI: 10.1126/sciadv.aat5218, L. Fabregas Ibanez et al., DOI: 10.5194/mr-1-209-2020

01-Feb-2022 15:21:21

1. Distance distributions

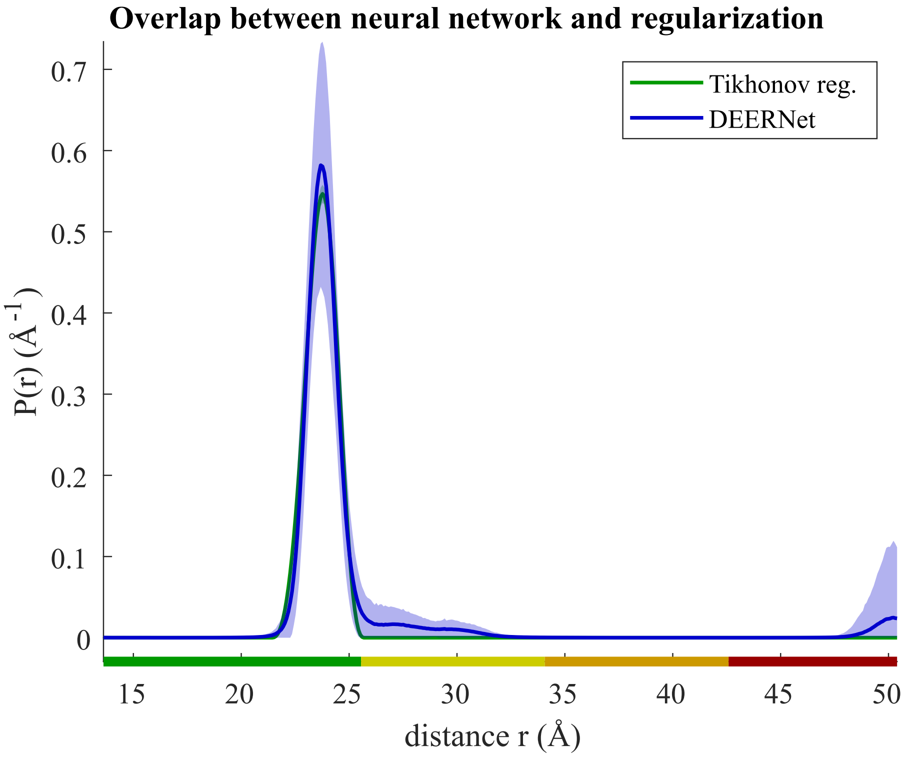

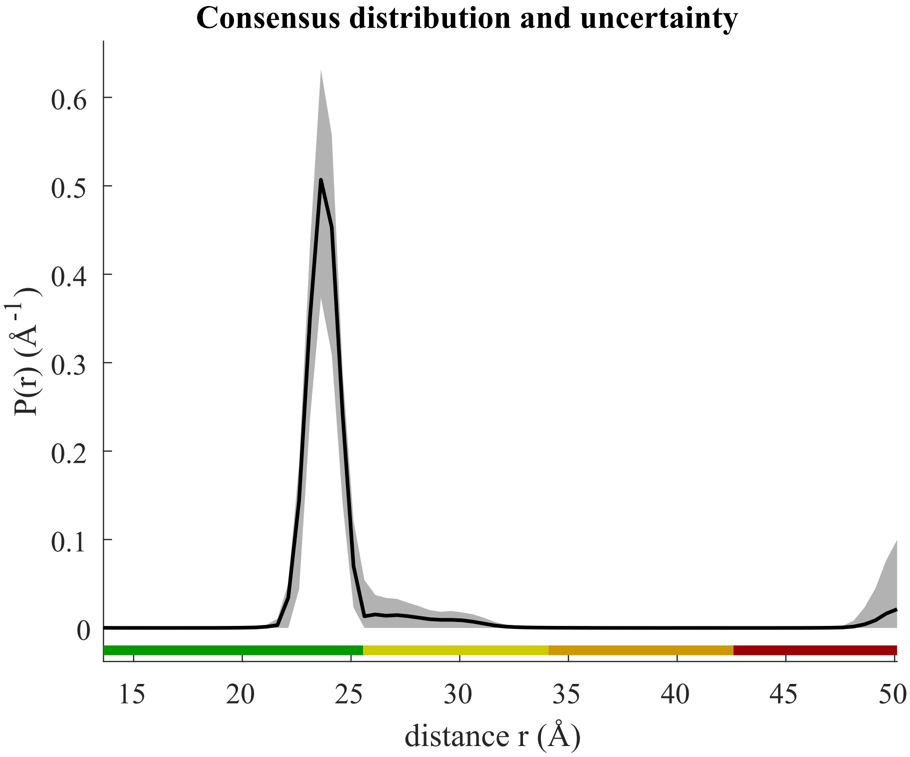

2. Fits of time-domain data

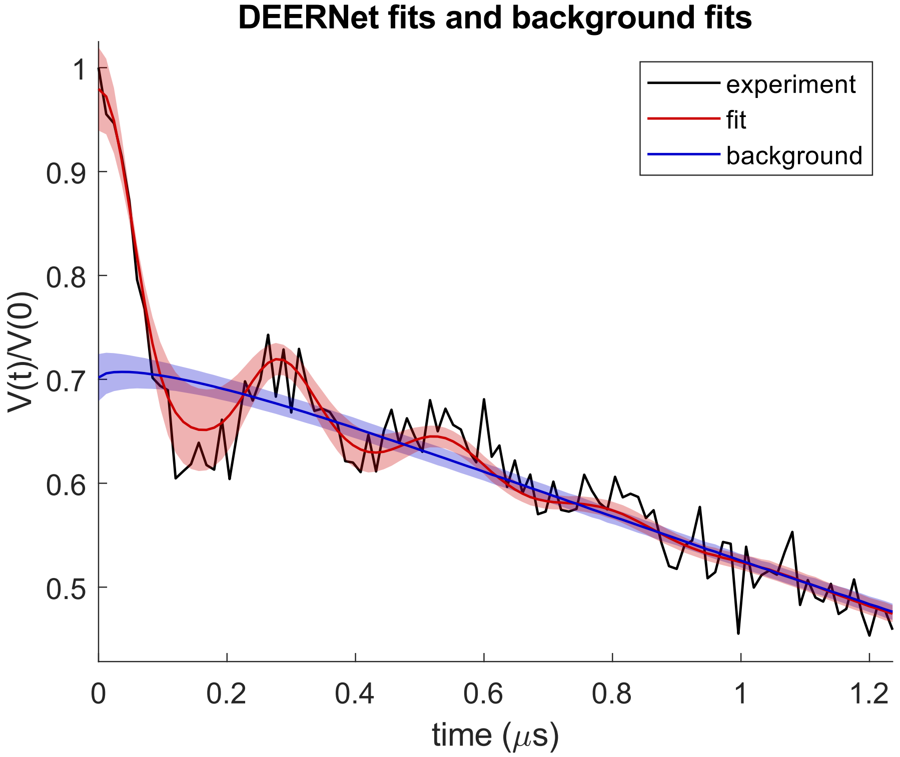

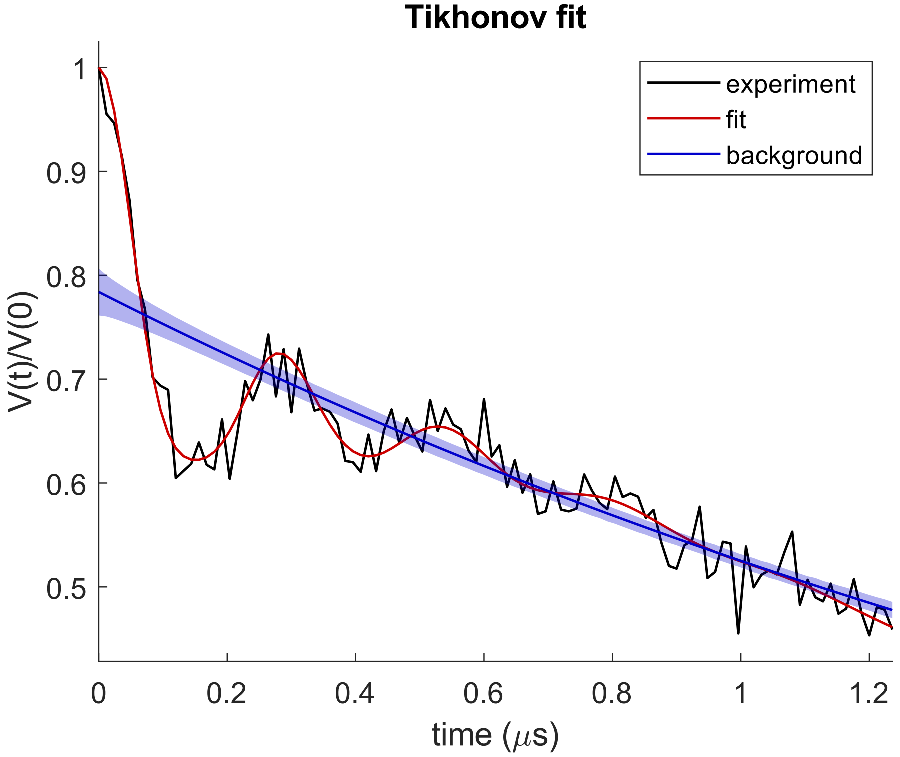

3. Experimental and processing parameters

DEERNet background not maximum at zero time. Long distances very uncertain.

Time increment: 12 ns

Maximum time: 1236 ns

Zero time: 6 ns

Phase: 0.0 degree

Noise estimates normalized to maximum signal

From imaginary part: 0.02328

From DEERNet fit: 0.02340

From Tikhonov fit: 0.02067

Modulation depth: 0.298

Signal-to-noise ratio: 12.7 (w.r.t. modulation)

Ensemble of 24 neural networks

Background separation by neural network

Regularization parameter by best overlap with neural network solution

Regularization parameter used: 1.00

Reg. par. initial estimate by L-curve corner: 3.98

Overlap between DEERNet and regularization solutions: 0.885

Mean distance: 23.8 Å

Distance standard deviation: 0.1 Å

Full data set in Matlab format: C:\Users\ka44\Documents\OneDrive - University of St Andrews\StAndrews\Work\BEB\Projects\GB1\GB1_Nanomolar\GB1_nM_data\Cu_NO\Titrations_Processing_2022\CDA\100nM_190411\190615_KAq108.3_RIDME_5_200_200000_by_5000_consensus_DEER_analysis.mat

Distance distributions in text format: C:\Users\ka44\Documents\OneDrive - University of St Andrews\StAndrews\Work\BEB\Projects\GB1\GB1_Nanomolar\GB1_nM_data\Cu_NO\Titrations_Processing_2022\CDA\100nM_190411\190615_KAq108.3_RIDME_5_200_200000_by_5000_consensus_DEER_distribution.dat

Fit and background in text format: C:\Users\ka44\Documents\OneDrive - University of St Andrews\StAndrews\Work\BEB\Projects\GB1\GB1_Nanomolar\GB1_nM_data\Cu_NO\Titrat

ions_Processing_2022\CDA\100nM_190411\190615_KAq108.3_RIDME_5_200_200000_by_5000_consensus_DEER_fit.dat

DEER analysis report on dataset 190609_KAq107.3_RIDME_5_200_200000_by_5000

DEERNet Spinach SVN Rev 5501 and DeerLab 0.9.1 Tikhonov regularization

ComparativeDEERAnalyzer

see: S. G. Worswick et al., DOI: 10.1126/sciadv.aat5218, L. Fabregas Ibanez et al., DOI: 10.5194/mr-1-209-2020

01-Feb-2022 15:12:45

1. Distance distributions

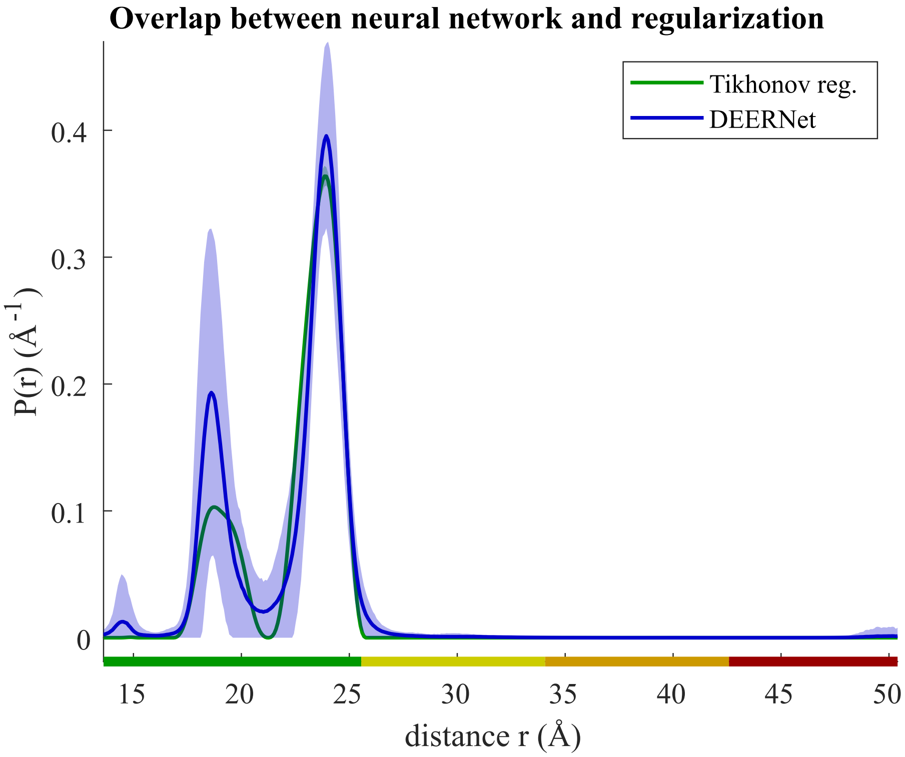

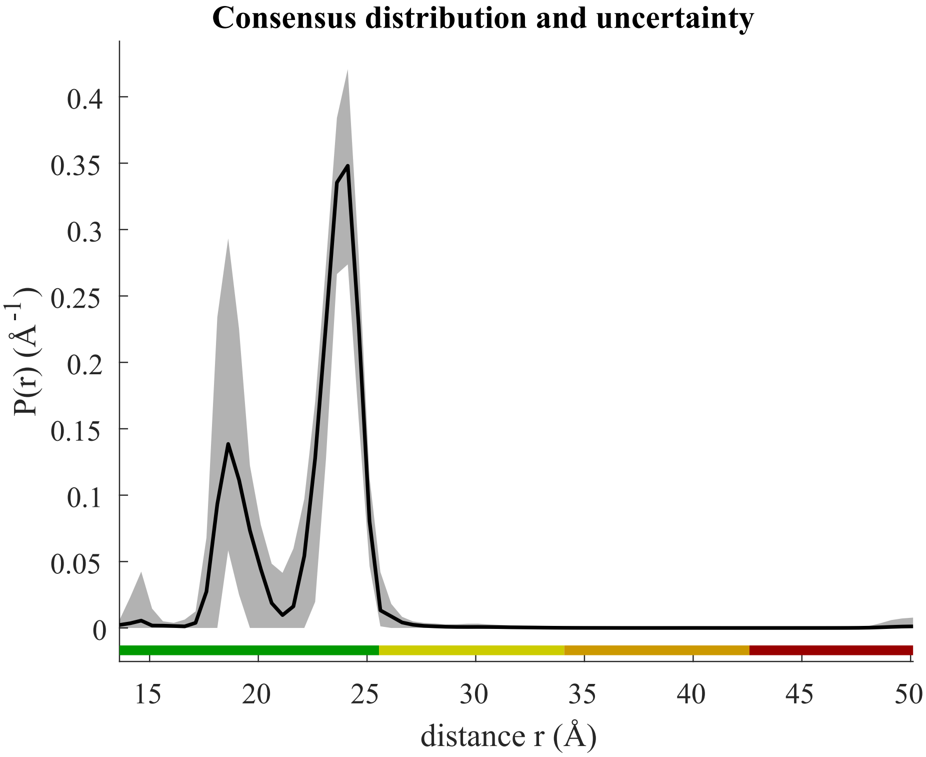

2. Fits of time-domain data

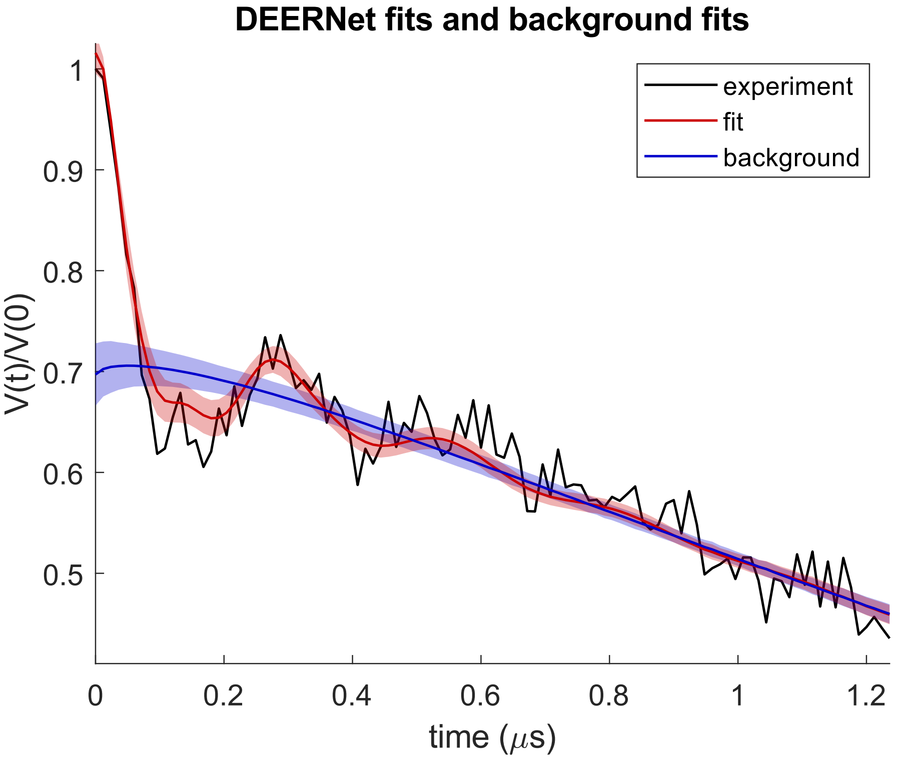

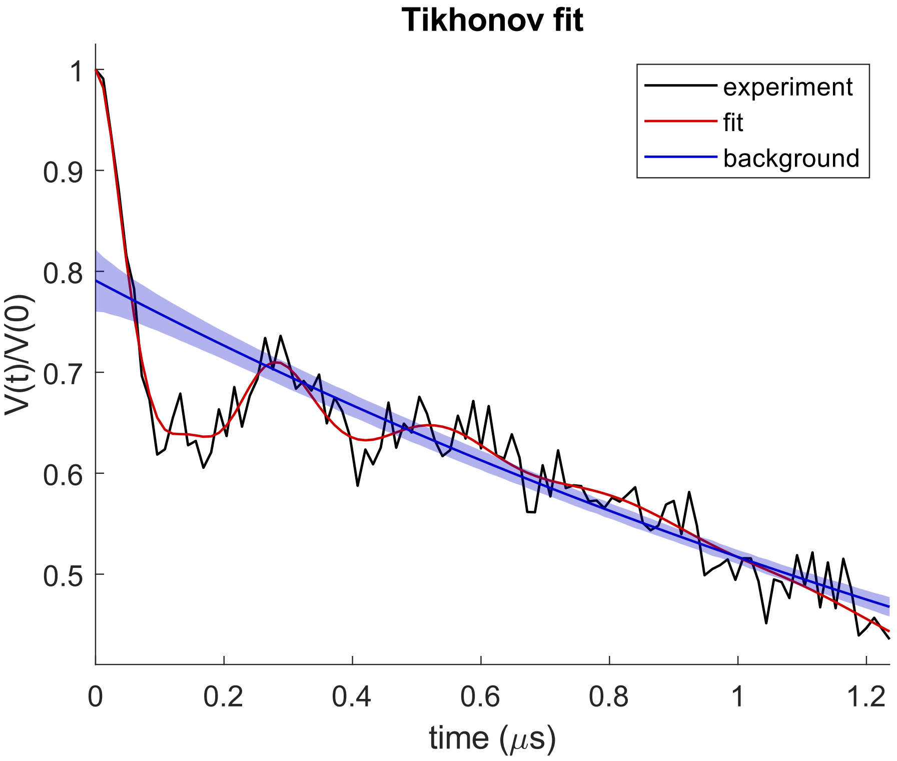

3. Experimental and processing parameters

DEERNet background not maximum at zero time. Long distances very uncertain.

Time increment: 12 ns

Maximum time: 1236 ns

Zero time: 5 ns

Phase: -0.4 degree

Noise estimates normalized to maximum signal

From imaginary part: 0.02508

From DEERNet fit: 0.02447

From Tikhonov fit: 0.02080

Modulation depth: 0.303

Signal-to-noise ratio: 12.4 (w.r.t. modulation)

Ensemble of 24 neural networks

Background separation by neural network

Regularization parameter by best overlap with neural network solution

Regularization parameter used: 1.25

Reg. par. initial estimate by L-curve corner: 5.01

Overlap between DEERNet and regularization solutions: 0.868

Mean distance: 23.5 Å

Distance standard deviation: -0.8 Å

Full data set in Matlab format: C:\Users\ka44\Documents\OneDrive - University of St Andrews\StAndrews\Work\BEB\Projects\GB1\GB1_Nanomolar\GB1_nM_data\Cu_NO\Titrations_Processing_2022\CDA\100nM_190411\190609_KAq107.3_RIDME_5_200_200000_by_5000_consensus_DEER_analysis.mat

Distance distributions in text format: C:\Users\ka44\Documents\OneDrive - University of St Andrews\StAndrews\Work\BEB\Projects\GB1\GB1_Nanomolar\GB1_nM_data\Cu_NO\Titrations_Processing_2022\CDA\100nM_190411\190609_KAq107.3_RIDME_5_200_200000_by_5000_consensus_DEER_distribution.dat

Fit and background in text format: C:\Users\ka44\Documents\OneDrive - University of St Andrews\StAndrews\Work\BEB\Projects\GB1\GB1_Nanomolar\GB1_nM_data\Cu_NO\Titrat

ions_Processing_2022\CDA\100nM_190411\190609_KAq107.3_RIDME_5_200_200000_by_5
000_consensus_DEER_fit.dat
